# Continuous Flow Approach for Benzylic Photo-oxidations Using Compressed Air

**DOI:** 10.1021/acs.oprd.4c00213

**Published:** 2024-07-24

**Authors:** Ruairi Bannon, Gary Morrison, Megan Smyth, Thomas S. Moody, Scott Wharry, Philippe M. C. Roth, Guillaume Gauron, Marcus Baumann

**Affiliations:** † School of Chemistry, Science Centre South, University College Dublin, Dublin D04 N2E5, Ireland; ‡ Technology Department, Almac Sciences, Craigavon BT63 5QD, U.K.; § Arran Chemical Company, Monksland Industrial Estate, Roscommon N37 DN24, Ireland; ∥ Corning Reactor Technologies, Corning SAS, 7 bis Avenue de Valvins, CS 70156 Samois sur Seine, 77215 Avon Cedex, France

**Keywords:** photooxidation, continuous flow, gas−liquid system, aerobic oxidation

## Abstract

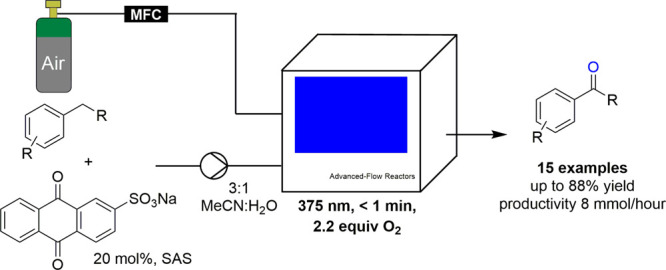

A continuous flow approach for the aerobic photo-oxidation of benzylic substrates to ketone and aldehyde products is presented. The resulting process exploits UV-A LEDs (375 nm) in combination with a Corning AFR reactor that ensures effective gas–liquid mixing and leads to short residence times of 1 min. A variety of benzylic substrates are converted to their corresponding carbonyl products, and scalability is demonstrated to produce multigram quantities of products within a few hours. Overall, this continuous flow approach offers several improvements over alternative oxidation methods due to the combined use of air as an oxidant and SAS (sodium anthraquinone-2 sulfonate) as a water-soluble photocatalyst. The use of greener and safer conditions together with process intensification principles renders this flow approach attractive for further industrial applications.

## Introduction

New and improved oxidation processes are highly sought after within academia and industry.^1^ Of specific interest is the direct oxidation of benzylic positions as this constitutes a key approach to manufacturing carbonyl-based fine chemicals,^2^ however, traditional methods tend to use stoichiometric oxidants generating equimolar amounts of waste such as MnO_2_^3^ or hypervalent iodine.^4,5^ Direct utilization of oxygen gas as an oxidant is a much more desirable and sustainable method for carrying out oxidations as minimal amounts of low molecular weight waste are generated.^6−8^ Moreover, harnessing oxygen directly from the air rather than using pure oxygen gas is an attractive option, especially at the industrial scale.^9^ This offers safer reaction conditions, as excess oxygen is prevented, reducing the risk of fire by solvent ignition. Previous work using compressed air as an oxygen supply demonstrated the significant improvement achievable with ultrafine bubble technology, allowing straightforward supersaturation of the reaction solution.^10^

Continuous flow technology is a valuable approach for photochemical reactions due to the short path length of light (i.e., the distance between light source and reactant solution) and narrow reactor channels that allow for full penetration of light.^11^ Recent years have witnessed a steady increase in reports detailing both home-built set-ups as well as, commercial flow photoreactor platforms which have the advantage of being standardized.^12^ Among the latter, Corning’s advanced-flow reactor (AFR) has been exploited for several continuous photochemical transformations with benefits including the rapid screening of various tunable wavelengths and intensities. The AFR is based on a plate reactor that provides high heat and mass transfer due to the development of the fluidic module^13^ containing heart-shaped cells for improved mixing.^14^ The combination of continuous flow processing with light to provide photons as the driving force for aerobic oxidation reactions is highly desirable for the efficient execution of benzylic photooxidations.^15−17^

Benzylic photooxidations have been performed using a range of metal-containing catalysts such as tetra-butylammonium decatungstate (TBADT),^18^ which can oxidize both activated and unactivated C–H bonds. Organic dyes such as Eosin Y^17^ and Rose Bengal^19^ have also been shown to facilitate photooxidations. Typically, these methods use pure O_2_. Quinones are well-known photo-organocatalysts^20−22^ including sodium anthraquinone-2 sulfonate (SAS) that has been used as a hydrogen atom acceptor to perform mild oxidations generating alcohols, aldehydes, and ketones with negligible overoxidation to carboxylic acids.^23−25^ Alternative counterions such as tetrabutylammonium have been reported along with metal salt additives (e.g., Co(acac)_2_) which solubilize anthraquinone catalysts in organic solvents.^26^ When performed in batch, some direct photooxidation of benzylic C–H bonds using anthraquinones required relatively long reaction times up to 24 h.^26^ Based on the prevalence of flow-based oxidation reactions with contributions from our group^27^ and others,^28^ we wished to address this issue, and set out to develop a readily scalable continuous photooxidation process for benzylic substrates under aerobic conditions using compressed air. SAS was thereby chosen as the photocatalyst due to its water-solubility, which allows the use of water as a green cosolvent as well as simple removal of the catalyst during extractive workup.

## Results and Discussion

**Scheme 1:**
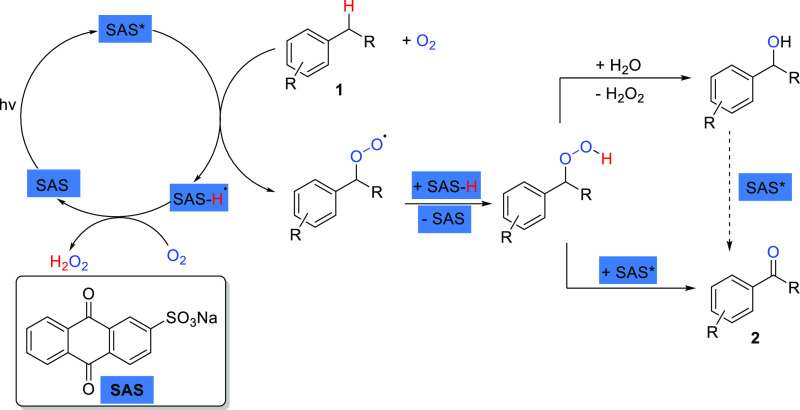
Photocatalytic Cycle of Sodium Anthraquinone-2 Sulfonate (SAS)

The catalytic cycle of SAS has been studied previously,^23^ showing that the photocatalyst requires oxygen to regenerate from its reduced state once it has abstracted a hydrogen atom from the benzylic position of the substrate. Therefore, the amount of oxygen in the system must consider the need for oxygen to reform the ground state of SAS. As shown in Scheme 1, it is possible for an alcohol side product to form through fragmentation of the peroxide intermediate, which can then react with further SAS to form the desired ketone product via hydrogen atom abstraction.

Diphenylmethane (**1a**) was chosen as the model substrate during the initial investigations. The standard conditions used 20 mol % of SAS, meaning that due to its catalytic cycle, 0.2 equiv of oxygen would be required for its regeneration, whereas the remaining oxygen is utilized for the formation of benzophenone **2a**.

Under standard conditions, full conversion of diphenylmethane was achieved with a ^1^H NMR yield of benzophenone of 92% (Table 1, entry 1). When the liquid flow rate was increased to 1.6 mL/min, the gas flow rate had to also be increased to maintain 2.2 equiv of oxygen in the system (entry 2). This reduced the residence time, which caused a reduction in yield for **2a**. When investigating the effect of a shorter wavelength of light (i.e., 340 nm instead of 375 nm) a lower conversion and yield of the ketone product was observed (entry 3). Although the UV–vis spectrum of SAS indicates absorbance between 300 and 400 nm with the maximum absorbance at 328 nm (Figure S1) this outcome is likely caused by the diminished intensity of the 340 nm LEDs compared to those emitting at 375 nm (2.2 and 46 W, respectively). Reducing the amount of both oxygen and catalyst loading still gave high substrate conversions, but in both cases, the yield for the ketone product was below that seen under the standard conditions (entries 4 and 5). Performing the reaction without a stream of air resulted in only 10% substrate conversion with no ketone product observed, instead a dimer of diphenylmethane was observed (entry 6) (the solution was degassed and only the liquid pump was turned on). In the final two control experiments without light or photocatalyst, no reaction took place (entries 7 and 8).

**Table 1:**
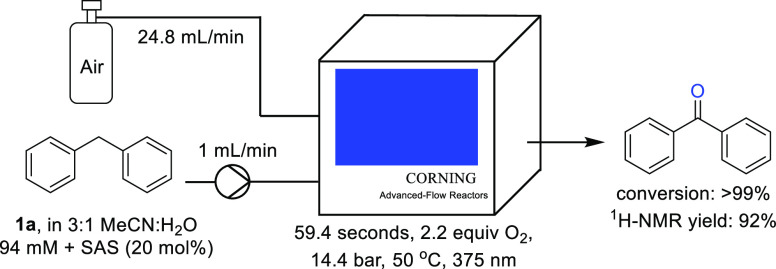
Continuous Flow Photooxidation: Optimization and Control Experiments

entry	deviations from standard conditions	conversion^a^	yield^a^
**1**		>99%	92%
**2**	1.6 mL/min, 39.8 mL/min air, *t*_Res_ 37.2 s	97%	77%
**3**	340 nm	15%	10%
**4**	1.2 equiv of O_2_	98%	60%
**5**	10 mol % catalyst loading	96%	78%
**6**	no air	10%^b^	0%
**7**	no light	0%	ND
**8**	no photocatalyst	0%	ND

a Determined via ^1^H NMR using 1,3,5-trimethoxybenzene as internal standard.

b Substrate dimer observed. ND – not detected.

Having identified suitable conditions for the continuous photooxidation of diphenylmethane in a short residence (i.e., 92% yield in ∼1 min), we wished to test some additional substrates under similar conditions. This effort was driven by earlier reports using SAS as a catalyst stating that modifications in the reaction time and catalyst loading are often required.^23^

### Effect of Liquid Flow Rate

Altering the flow rate of the substrate solution not only results in the change of material being processed but also necessitates adjusting the amount of available gas to ensure delivery of the intended 2.2 equiv of oxygen. Under the previously established standard conditions, the photooxidation of substrate **1g** showed complete conversion with a product yield of 59% (Table 2, entry 1). A shorter residence time was investigated by increasing both liquid and gas flow rates; however, this showed little effect on the yield (entry 2). For this reason, the faster flow rate of 1.6 mL/min was preferable for this substrate due to a higher throughput with similar yields being achieved because of effective micromixing. On the contrary, when treating substrate **1e** under photochemical conditions, the initially established longer residence time led to an increase of conversion and yield by ca. 20% compared to the shorter residence time (Table 2, entries 3 and 4). With the starting material remaining, the product solution was recycled once, and the product yield increased further by 16% (entry 5).

**Table 2:**
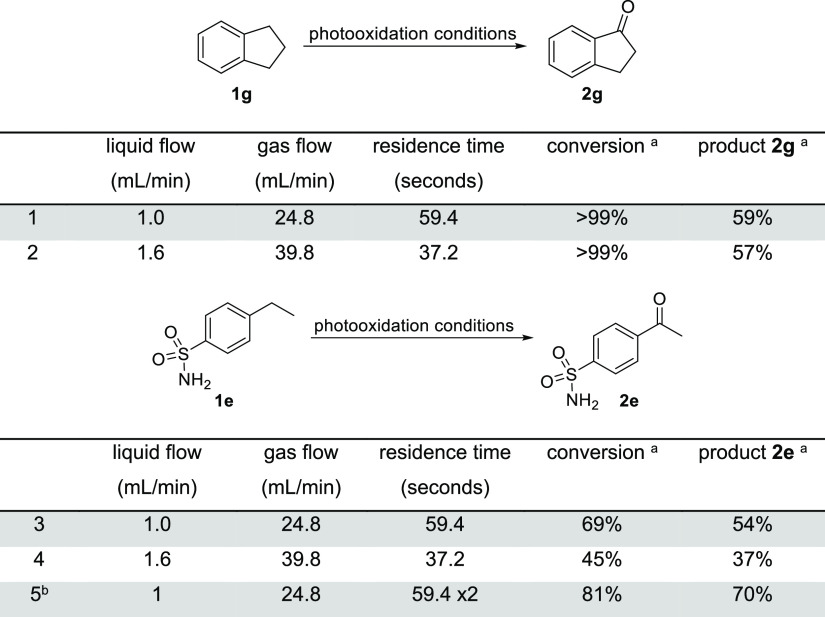
Altering Liquid and Gas Flow Rates for Substrates **1g** and **1e**^a^

a Photooxidation conditions: liquid flow rate, gas flow rate, and residence time as stated above, 2.2 equiv O_2_, 20 mol % SAS, 3:1 MeCN: H_2_O, 375 nm, 14.4 bar, 50 °C. ^a1^H NMR yield using 1,3,5-trimethoxybenzene as the internal standard. ^b^Reaction mixture was recycled once.

### Effect of Increased Catalyst Loading

SAS is known to oxidize tolyl groups to the corresponding aldehydes,^23^ however, when using 20 mol % of SAS similarly to our standard conditions, lower yields for aldehyde **2n** were obtained than observed when synthesizing ketone targets. As seen in Table 3, longer residence times did not generate more product, although the substrate conversion increased (entries 1 and 2) which indicates that undesired side reactions dominate. However, when increasing the amount of catalyst to 40 mol % and adjusting the solvent mixture to ensure full solubility (i.e., 1:1 MeCN:H_2_O) a notable increase in product yield was observed (entry 3).

**Table 3:**
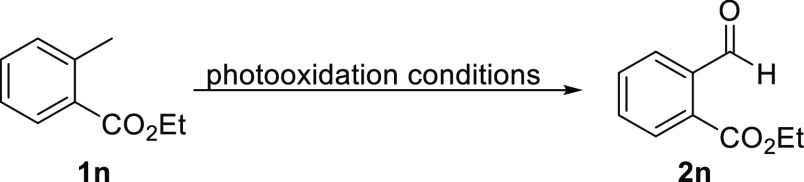
Altering Liquid Flow Rates, Gas Flow Rates, and Catalyst Loading for Substrate **1n**

	catalyst loading (mol %)	liquid flow (mL/min)	gas flow (mL/min)	residence time (s)	conversion^a^	product **2h**^a^
1^b^	20	1.6	39.8	37.2	38%	27%
2^b^	20	1.0	24.8	59.4	50%	27%
3^c^	40	1.6	39.8	37.2	77%	40%

a ^1^H NMR yield using 1,3,5-trimethoxybenzene as internal standard.

b Liquid flow rate, gas flow rate, and residence time as stated above, 2.2 equiv O_2_, 20 mol % SAS, 3:1 MeCN: H_2_O, 375 nm, 14.4 bar, 50 °C.

c Liquid flow rate, gas flow rate, and residence time as stated above, 2.2 equiv O_2_, 20 mol % SAS, 1:1 MeCN: H_2_O, 375 nm, 14.4 bar, 50 °C.

**Scheme 2:**
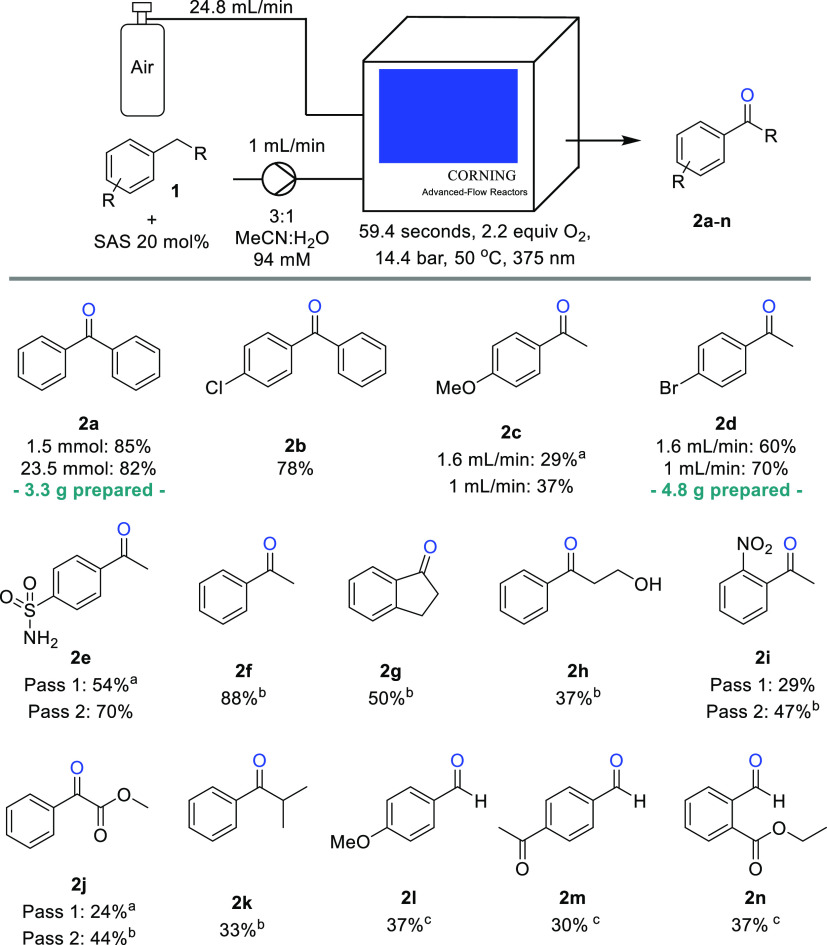
Photooxidation Substrate Scope ^1^H NMR yield using 1,3,5-trimethoxybenzene as internal standard. ^b^1.6 mL/min liquid, 39.8 mL/min air, 14.4 bar, 37.2 s. ^c^1.6 mL/min liquid, 39.8 mL/min air, 14.4 bar, 37.2 s, 40 mol % SAS.

With the learnings from the initial substrate optimization studies, we next embarked on establishing the scope of this continuous aerobic photooxidation process. As indicated in Scheme 2, doubly benzylic substrates such as **2a** and **2b** worked best with a longer residence time of ca. 1 min. Alkyl benzenes generally were effective as substrates; however, electron-rich systems gave slightly lower yields (i.e., **2c**) even when both flow rates were decreased. Notably, the bromide substituent in product **2d** was readily tolerated, and a slightly improved yield was achieved when increasing the residence time (i.e., 60 vs 70%).

Other alkyl benzenes afforded the desired ketone products (**2e**–**i**) in good yields and within short residence times (37 s). In most cases, incomplete substrate conversion along with the intermediate secondary alcohol product was observed. The oxidation is thereby selective for the benzylic position over additional oxidation-sensitive moieties such as in the case of **2h**, where no aldehyde formation was observed. This is in agreement with prior batch oxidation procedures using SAS where oxidation of aliphatic alcohols occurs more slowly (i.e., over 24 h).^23^ Performing this photooxidation in continuous flow mode with very short residence times accelerates the desired reaction while retaining the selectivity for the benzylic position. In cases where significant amounts of substrate remained, a second pass of the crude mixture through the photoreactor setup leads to increased yields as seen for products **2e**, **2i**, and **2j**. In the case of product **2k**, the C–C bond cleavage giving benzaldehyde as a side product (∼40%) was observed at 375 nm. Alternative wavelengths (340, 395, and 422 nm) were trialed to reduce the amount of C–C bond cleavage, however, this was not successful with benzaldehyde still being the major product in all cases (Table S1). Further investigations subjecting either the ketone product or the corresponding benzylic alcohol to the reaction conditions indicated that the fragmentation arises from the latter (Scheme S1). As stated previously, when synthesizing aldehydes from substituted methylbenzenes, 40 mol % of the catalyst is preferable yielding aldehydes **2l**–**2n** in modest yields, which demonstrates the lower reactivity of these substrates.

Additionally, our efforts targeted the scale-up of this flow process to generate multigram quantities of products **2a** and **2d** over periods of 4 and 6 h, respectively. Pleasingly, the small-scale yields were reproduced, showing that the process is stable under steady-state conditions (see Scheme 2). In both cases, small amounts of remaining substrate and the corresponding secondary alcohol (ca. 5–10%) were found in the crude material which is consistent with our earlier observations.

**Scheme 3:**
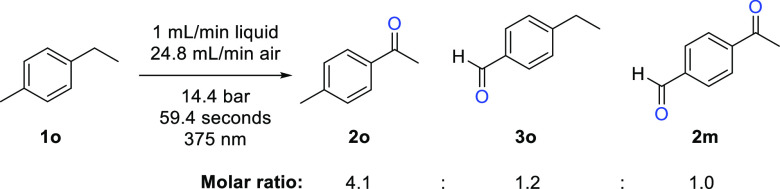
Photooxidation of 1-Ethyl-4-methylbenzene **1o** Ratio was determined using ^1^H NMR using 1,3,5-trimethoxybenzene as internal standard.

Due to the difference in reactivity for the formation of ketone and aldehyde products, we decided to also employ substrates such as 1-ethyl-4-methylbenzene **1o** under the photooxidation conditions as seen in Scheme 3. This study revealed that ketone **2o** was indeed the major product formed whereas aldehyde **3o** and the double oxidation product **2m** were obtained as minor products. This outcome was reproduced when varying the reaction conditions and accounted for ca. 45% of the crude product, indicating that different partially oxidized intermediates were generated as well.

## Conclusions

A scalable flow process for the photooxidation of various alkylbenzene substrates to their corresponding ketone and aldehyde products was developed using water-soluble SAS as a photocatalyst with a residence time of less than 60 s. Near stoichiometric amounts of oxygen were delivered via compressed air that was used as a safer alternative to pure oxygen gas. Together with the effective gas–liquid mixing achieved using a Corning AFR photoreactor this approach clearly enhances the green credentials of the overall process. The effect of varying liquid and gas flow rates, catalyst loading, and the wavelength of the LED source was studied across a variety of benzylic substrates demonstrating that this green oxidation method is general while showing differentiated reactivity. The resulting flow process was operated for up to 6 h which delivered multigram quantities of product under steady state conditions. Overall, the data show that aerobic photooxidation reactions can be intensified under continuous flow conditions using a Corning AFR reactor which is expected to facilitate further studies and applications in industrial settings.

## Experimental Procedure

Unless stated otherwise, the reagent was dissolved in a mixture of acetonitrile and water (3:1, 94 mM) containing sodium anthraquinone sulfonate (SAS, 20 mol %). Compressed air (24.8 mL/min) and solvent (1 mL/min) were pumped through the reactor plate (volume 2.7 mL) to allow the system to reach the desired pressure (14.4 bar) and temperature (50 °C). Once the system was at the desired conditions, the LEDs (375 nm) were switched on, and the reaction solution was pumped through the reactor plate. Two plate volumes were discarded before collecting the product stream, ensuring the reaction was at a steady state. Once collected, acetonitrile was evaporated under pressure, giving a mixture of the product and water. The product was extracted by using ethyl acetate (3 × 20 mL). The organic layers were then combined and washed with saturated sodium sulfite solution to quench hydrogen peroxide formation and finally with brine. The organic layer was dried using sodium sulfate, and ethyl acetate was removed under vacuum to yield the crude product. The desired product was isolated via column chromatography. For alternative reaction conditions for specific substrates, this information is available in the Supporting Information.

Due to being a gas–liquid system, the total flow rate and residence time are defined as
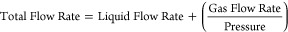

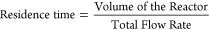


Total flow rate, liquid flow rate, and gas flow rate are given in units of mL/min. Pressure is given in bar, volume of the reactor in mL, and residence time is given in minutes.

## Supporting Information Available

Image of the reactor setup, alternative reaction conditions, UV–vis spectrum of sodium-anthraquinone-2-sulfonate, copies of ^1^H and ^13^C NMR spectra (PDF)
