# Supplementary material for: Continuous Flow Approach for Benzylic Photo-oxidations Using Compressed Air
Source: Org Process Res Dev. 2024 Jul 24;28(8):3307–12. doi: 10.1021/acs.oprd.4c00213 (PMC11334174; doi:10.1021/acs.oprd.4c00213)

A Continuous Flow Approach for Benzylic Photooxidations using Compressed Air

Supporting Information.

Ruairi Bannon,[a] Gary Morrison,[b] Megan Smyth,[b] Thomas S. Moody,[b,c] Scott Wharry,[b] Philippe M. C. Roth,[d] Guillaume Gauron,[d] Marcus Baumann [a,*]

[a] University College Dublin, School of Chemistry, Science Centre South, D04N2E5, Ireland

[b] Technology Department, Almac Sciences, Craigavon BT63 5QD, United Kingdom

[c] Arran Chemical Company, Monksland Industrial Estate, Roscommon N37 DN24, Ireland

[d] Corning Reactor Technologies, Corning SAS, 7 bis Avenue de Valvins, CS 70156 Samois sur Seine, 77215 Avon Cedex, France

Corresponding author: marcus.baumann@ucd.ie

Table of Contents:

Materials and Methods..2
Experimental Procedure..3
Spectroscopic Data..5
References..8
NMR Data..9

Materials and Methods

Unless otherwise stated, all solvents were purchased from Fisher Scientific or Sigma Aldrich and used without further purification. Substrates and reagents were purchased from Alfa Aesar, Fluorochem or Sigma Aldrich and used as received. Compressed air was purchased from BOC and used as received.

^{1}H-NMR spectra were recorded on 400 MHz instruments and are reported relative to residual solvent: $CDCl_3$ (δ 7.26 ppm) or TMS (δ 0 ppm) in cases where residual solvent was not clearly visible. ^{13}C-NMR spectra were recorded on the same instruments (100 MHz or 125 MHz) and are reported relative to the corresponding solvent: $CHCl_3$ (δ 77.16 ppm).

Data for ^{1}H-NMR are reported as follows: chemical shift (δ/ ppm) (integration, multiplicity, coupling constant (Hz)). Multiplicities are reported as follows: s = singlet, d = doublet, t = triplet, q = quartet, sext = sextet, sept = septet, m = multiplet. Data for ^{13}C-NMR are reported in terms of chemical shift (δ/ ppm) and multiplicity (C, CH, CH_2 or CH_3).

Photooxidation reaction was carried out using a Corning© Advanced-Flow™ photoreactor. Samples were irradiated by 2 LED panels emitting UV light (375 nm unless stated overwise) onto a microplate reactor (2.7 mL). LEDs were chilled using a Huber chilling unit and a separate Huber chilling unit was used to control the temperature of the fluidic plate. Residence time was determined by liquid flow rate (mL/min), gas flow rate (mL/min), and pressure (bar) set by a back pressure regulator (BPR). Air was pumped into the system controlled by a mass flow controller (MFC) to achieve accurate flow rate.

Gas chromatography analysis was performed on an Agilent 8860 system with a HP-5 (30 m x 320 μm x 0.25 μm) column installed. Set point was set to 250 °C and the FID detector was at 300 °C. Oven temperature was set 60 °C, heated at 10 °C/min until 200 °C and held for 1 minute until a further temperature ramp of 25 °C/min until 250 °C and held for 2 minutes. Split ratio set to 10:1 and a pressure of 8.6 psi. Flow is set 1.67 mL/min with air flow set to 400 mL/min, H_2 flow 40 mL/min and makeup flow 25 mL/min.

Experimental Procedure

Conditions A

Liquid Flow Rate 1 mL/min, Gas flow rate 24.8 mL/min (2.2 equivalents of O_2), BPR set to 14.4 bar, residence time 59.4 seconds, 3:1 MeCN:H_2O, catalyst loading 20 mol%.

Condition B

Liquid Flow Rate 1.6 mL/min, Gas flow rate 39.8 mL/min (2.2 equivalents of O_2), BPR set to 14.4 bar, residence time 37.2seconds, 3:1 MeCN:H_2O, catalyst loading 20 mol%.

Condition C

Liquid Flow Rate 1.6 mL/min, Gas flow rate 39.8 mL/min (2.2 equivalents of O_2), BPR set to 14.4 bar, residence time 37.2seconds, 1:1 MeCN:H_2O, catalyst loading 40 mol%.

The reagent was dissolved in acetonitrile: water (94 mM) as well as sodium anthraquinone sulfonate (SAS). The compressed air and the solvent were pumped through the reactor plate to allow the system to reach pressure and 50°C. Once system was at the desired conditions the LEDs (375 nm) were switched on the reaction solution was pumped through the reactor plate. Two plate volumes were discarded before collecting ensuring the reaction was at steady state. Once collected, acetonitrile was removed under pressure being left with organic product and water. Product was extracted using ethyl acetate (3 x 20 mL). Organic layers were then combined and washed with saturated sodium sulfite solution to quench hydrogen peroxide formed and finally with brine. Organic layer was dried using sodium sulfate and ethyl acetate was removed under vacuum to yield crude product. Desired ketone was isolated via column chromatography.

Reactor Set-up

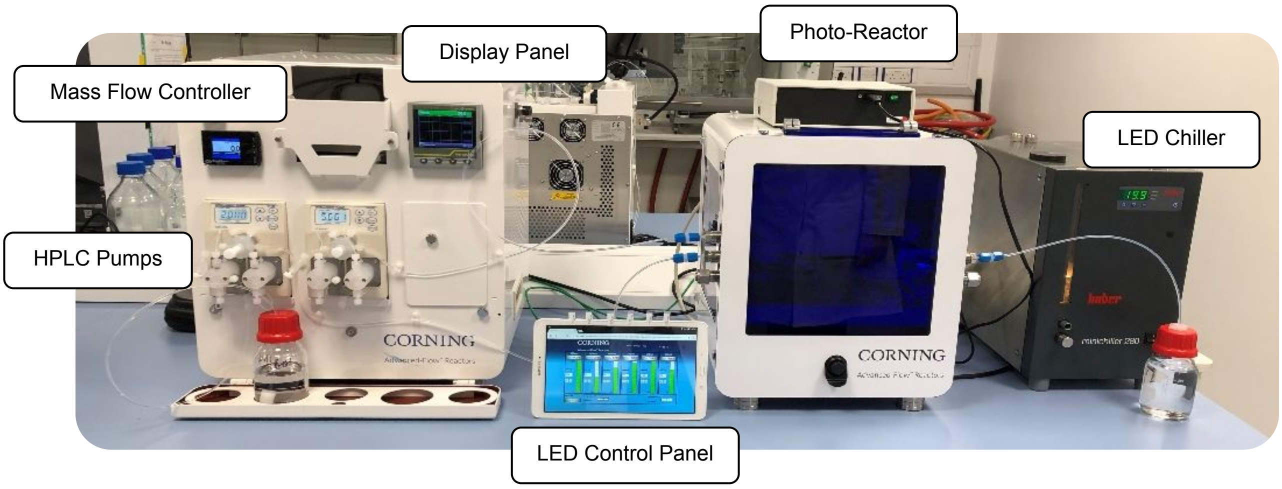

UV-Vis Spectrum.

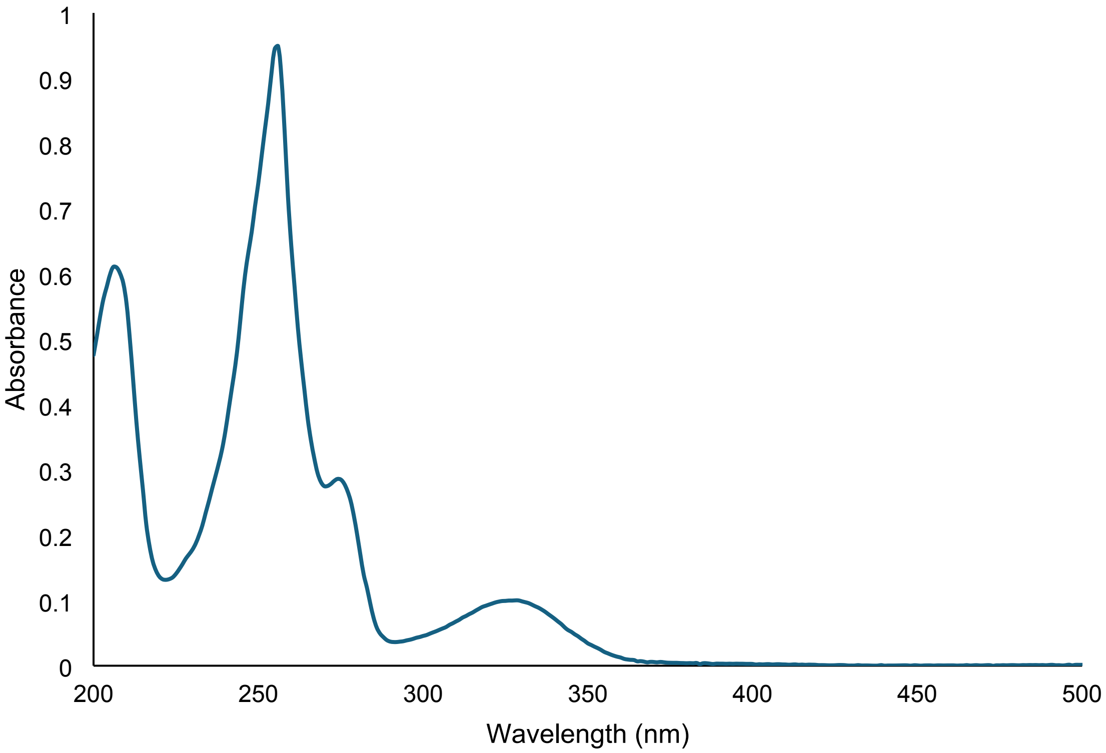

Figure S1 UV-Vis Spectrum of Sodium Anthraquinone-2-sulfonate. Ran in 3:1 MeCN:H_2O.

C-C bond cleavage on Isobutylbenzene 1k.

Table S1: Photooxidation of Isobutylbenzene **1k** at different wavelengths of light.

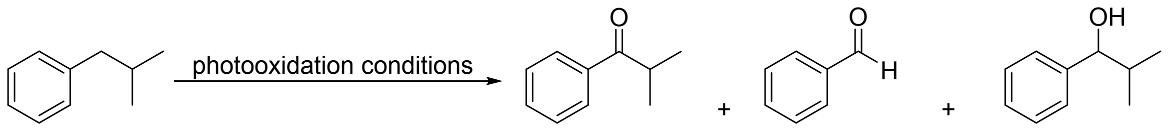

1k **2k** **3** **4k**

Wavelength	Starting Material **1k** [a]	Product **2k** [a]	Benzaldehyde **3** [a]	Alcohol **4k** [a]
340 nm	79%	6%	6%	2%
375 nm	2%	38%	40%	4%
395 nm	4%	39%	43%	5%
422 nm	51%	18%	22%	6%

Photooxidation conditions: 1.6 mL/min liquid, 39.8 mL/min air, 14.4 bar, 37.2 s, 50 °C, wavelength of light stated in table. [a] GC yields.

Spectroscopic Data

Benzophenone (2a): Synthesised by experimental **conditions A.**[1]

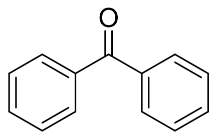

Chemical Formula: $C_{13}H_{10}O$
Exact Mass: 182.0732

Yield: 85% (234.4 mg, 1.28 mmol)

Scale up: 82% (3.3g, 18.1 mmol)

Appearance: White solid

^{1}H NMR (400 MHz, $CDCl_3$) δ= 7.84 – 7.76 (m, 4H), 7.62 – 7.56 (m, 2H), 7.52 – 7.45 (m, 4H). **^{13}C NMR** (101 MHz, $CDCl_3$) δ = 196.8 (C=O), 137.8 (CH), 132.5 (CH), 130.2 (CH), 128.4 (CH).

(4-chlorophenyl)(phenyl)methanone (2b): Synthesised by experimental **conditions A.**[2]

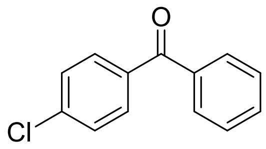

Chemical Formula: $C_{13}H_9ClO$
Exact Mass: 216.0342

Yield: 78% (239.1 mg, 1.1 mmol)

Appearance: White Solid

^{1}H NMR (400 MHz, $CDCl_3$) δ= 7.83 – 7.71 (m, 4H), 7.64 – 7.57 (m, 1H), 7.53 – 7.43 (m, 4H). **^{13}C NMR** (101 MHz, $CDCl_3$) δ= 195.6 (C=O), 139.0 (C), 137.4 (C), 136.0 (C), 131.6 (CH), 130.1 (CH), 128.8 (CH), 128.5 (CH).

4-Methoxyacetophenone (2c): Synthesised by experimental **conditions A.**[1]

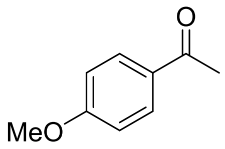

Chemical Formula: $C_9H_{10}O_2$
Exact Mass: 150.0681

Yield: 37% (79.1 mg, 0.52 mmol)

Appearance: Yellow Oil

^{1}H NMR (400 MHz, $CDCl_3$) δ= 7.96 – 7.91 (m, 2H), 6.96 – 6.90 (m, 2H), 3.87 (s, 3H), 2.55 (s, 3H). **^{13}C NMR** (101 MHz, $CDCl_3$) δ = 196.9 (**C**OCH_3), 163.6 (C), 130.7 (CH), 113.8 (CH), 55.6 (CO**C**H_3), 26.5 (CH_3).

4-Bromoacetophenone (2d): Synthesised by experimental **conditions A.**[1]

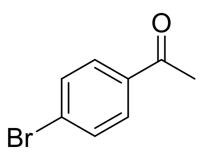

Chemical Formula: C_8H_7BrO
Exact Mass: 197.9680

Yield: 70% (195.7 mg, 0.98 mmol)

Scale up: 71% (4.8g, 24.1 mmol)

Appearance: White solid

^{1}H NMR (400 MHz, $CDCl_3$) δ= 7.86 – 7.73 (m, 2H), 7.64 – 7.56 (m, 2H), 2.58 (s, 3H, -CH_3). **^{13}C NMR** (101 MHz, $CDCl_3$) δ = 197.1 (C=O), 136.0 (C), 132.0 (CH), 130.0 (CH), 128.4 (C), 26.7 (CH_3).

4-Acetylbenzenesulfonamide (2e): Synthesised by experimental **conditions A.**[4] Reaction solution was collected and passed through the reactor a second time.

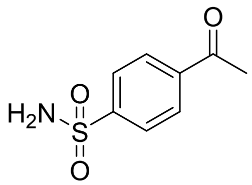

Chemical Formula: $C_8H_9NO_3S$
Exact Mass: 199.0303

Yield: 70% (196 mg, 0.98 mmol)

Appearance: White solid

^{1}H NMR (400 MHz, DMSO-D_6) δ= 8.21 – 8.03 (m, 2H), 8.04 – 7.88 (m, 2H), 7.54 (s, 2H, -NH_2), 2.63 (s, 3H, -CH_3). **^{13}C NMR** (101 MHz, DMSO-D_6) δ =197.4(C=O), 147.7 (C), 139.0 (C), 128.9 (CH), 126.0 (CH), 27.0 (CH_3).

Acetophenone (2f): Synthesised by experimental **conditions B.**[1]

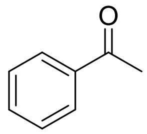

Chemical Formula: C_8H_8O
Exact Mass: 120.0575

Yield: 88% (159.2 mg, 1.32 mmol)

Appearance: Pale yellow oil

^{1}H NMR (400 MHz, $CDCl_3$) δ= 7.99 – 7.92 (m, 2H), 7.61 – 7.51 (m, 1H), 7.50 – 7.43 (m, 2H), 2.61 (s, 3H, -CH_3). **^{13}C NMR** (101 MHz, $CDCl_3$) δ = 198.3 (C=O), 137.3 (CH), 133.2 (CH), 128.7 (CH), 128.4 (CH), 26.73 (CH_3).

2,3-Dihydro-1H-inden-1-one (2g): Synthesised by experimental **conditions B.**[3]

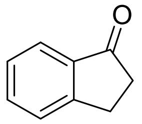

Chemical Formula: C_9H_8O
Exact Mass: 132.0575

Yield: 50% (98.8 mg, 0.75 mmol)

Appearance: Pale yellow oil

^{1}H NMR (400 MHz, $CDCl_3$) δ= 7.75 (d, *J* = 7.7 Hz, 1H), 7.58 (td, *J* = 7.4, 1.3 Hz, 1H), 7.47 (dt, *J* = 7.7, 1.0 Hz, 1H), 7.41 – 7.31 (m, 1H), 3.19 – 3.08 (m, 2H), 2.72 – 2.63 (m, 2H). **^{13}C NMR** (101 MHz, $CDCl_3$) δ= 207.2 (C=O), 155.3 (C), 137.2 (C), 134.7 (CH), 127.4 (CH), 126.8 (CH), 123.8 (CH), 36.3 (CH_2), 25.9 (CH_2).

3-Hydroxy-1-phenylpropan-1-one (2h): Synthesised by experimental **conditions B.**[3]

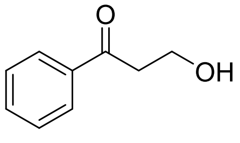

Chemical Formula: $C_9H_{10}O_2$
Exact Mass: 150.0681

Yield: 37% (84.2 mg, 0.56 mmol)

Appearance: Pale yellow oil

^{1}H NMR (400 MHz, $CDCl_3$) δ= 8.00 – 7.93 (m, 2H), 7.63 – 7.55 (m, 1H), 7.52 – 7.44 (m, 2H), 4.04 (q, J = 5.6 Hz, 2H), 3.23 (t, J = 5.3 Hz, 2H), 2.69 (t, J = 6.5 Hz, 1H). **^{13}C NMR** (101 MHz, $CDCl_3$) δ= 200.6 (C=O), 136.8 (C), 133.7 (CH), 128.8 (CH), 128.2 (CH), 58.2 (CH_2), 40.5 (CH_2).

1-(2-Nitrophenyl)ethan-1-one (2i): Synthesised by experimental **conditions B.**[5] Reaction solution was collected and passed through the reactor a second time.

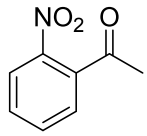

Chemical Formula: $C_8H_7NO_3$
Exact Mass: 165.0426

Yield: 47% (109.2 mg, 0.66 mmol)

Appearance: Brown oil

^{1}H NMR (400 MHz, $CDCl_3$) δ= 8.09 (dd, J = 8.3, 1.3 Hz, 1H), 7.72 (td, J = 7.5, 1.2 Hz, 1H), 7.60 (ddd, J = 8.2, 7.5, 1.5 Hz, 1H), 7.43 (dd, J = 7.5, 1.5 Hz, 1H), 2.55 (s, 3H). **^{13}C NMR** (101 MHz, $CDCl_3$) δ= 199.9 (C=O), 145.9 (C-NO_2), 138.2 (C), 134.4 (CH), 130.8 (CH), 127.47 (CH), 124.50 (CH), 30.28 (CH_3).

Methyl 2-oxo-2-phenylacetate (2j): Synthesis by experimental **conditions B.**[6] Reaction solution was collected and passed through the reactor a second time.

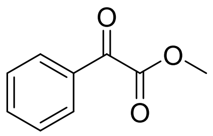

Chemical Formula: $C_9H_8O_3$
Exact Mass: 164.0473

Yield: 44% (161.6 mg, 0.98 mmol)

Appearance: Yellow Oil

^{1}H NMR (400 MHz, $CDCl_3$) δ= 8.06 – 7.97 (m, 2H), 7.71 – 7.63 (m, 1H), 7.52 (t, J = 7.8 Hz, 2H), 3.99 (s, 3H). **^{13}C NMR** (101 MHz, $CDCl_3$) δ= 186.2 (C=O), 164.2 (C=O), 135.2 (CH), 132.5 (C), 130.3 (CH), 129.1 (CH), 53.0 (CH_3).

Isobutylphenone (2k): Synthesised by experimental **conditions B.**[7]

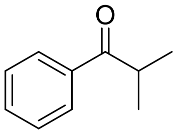

Chemical Formula: $C_{10}H_{12}O$
Exact Mass: 148.0888

Yield: 33% (73.4 mg, 0.5 mmol)

Appearance: Colourless oil

^{1}H NMR (400 MHz, $CDCl_3$) δ= 8.00 – 7.91 (m, 2H), 7.58 – 7.51 (m, 1H), 7.50 – 7.42 (m, 2H), 3.56 (hept, *J* = 6.8 Hz, 1H), 1.22 (d, *J* = 6.9 Hz, 6H). **^{13}C NMR** (101 MHz, $CDCl_3$) δ= 204.7 (C=O), 136.4 (C), 132.9 (CH), 128.7 (CH), 128.5 (CH), 35.5 (CH), 19.3 (CH_3).

4-Methoxybenzaldehyde (2l): Synthesised by experimental **conditions C.**[3]

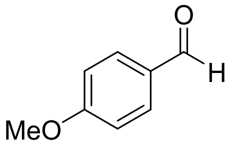

Chemical Formula: $C_8H_8O_2$
Exact Mass: 136.0524

Yield: 37% (76.3mg, 0.56 mmol)

Appearance: Yellow Oil

^{1}H NMR (400 MHz, $CDCl_3$) δ= 9.89 (s, 1H), 7.88 – 7.79 (m, 2H), 7.08 – 6.94 (m, 2H), 3.89 (s, 3H). **^{13}C NMR** (101 MHz, $CDCl_3$) δ= 191.0 (CHO), 164.8 (C), 132.1 (CH), 130.1 (C), 114.5 (CH), 55.7 (CH_3).

4-Acetylbenzaldehyde (2m): Synthesised by experimental **conditions C.**[6]

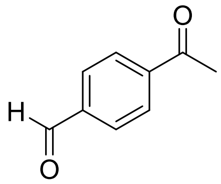

Chemical Formula: $C_9H_8O_2$
Exact Mass: 148.0524

Yield: 30% (62.8 mg, 0.42 mmol)

Appearance: White solid

^{1}H NMR (400 MHz, $CDCl_3$) δ= 10.11 (s, 1H), 8.15 – 8.06 (m, 2H), 8.03 – 7.94 (m, 2H), 2.66 (s, 3H). **^{13}C NMR** (101 MHz, $CDCl_3$) δ= 197.5 (C=O), 191.7 (C=O), 141.4 (C), 139.2 (C), 130.0 (CH), 129.0 (CH), 27.1 (CH_3).

Ethyl 2-formylbenzoate (2n): Synthesised by experimental **conditions C.**[8]

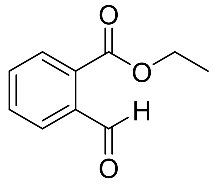

Chemical Formula: $C_{10}H_{10}O_3$
Exact Mass: 178.0630

Yield: 37%(99.5 mg, 0.56 mmol)

Appearance: Pale yellow oil

^{1}H NMR (400 MHz, $CDCl_3$) δ= 10.62 (s, 1H), 8.01 – 7.89 (m, 2H), 7.68 – 7.60 (m, 2H), 4.44 (q, J = 7.1 Hz, 2H), 1.42 (t, J = 7.1 Hz, 3H). **^{13}C NMR** (101 MHz, $CDCl_3$) δ= 192.3 (C=O), 166.5 (CHO), 137.1 (C), 133.0 (CH), 132.6 (C), 132.4 (CH), 130.5 (CH), 128.5 (CH), 62.1 (CH_2), 14.4 (CH_3).

References

[1] Nguyen, K.; Nguyen, V.; Tran, H.; Pham, P. Organo-photocatalytic C–H bond oxidation: an operationally simple and scalable method to prepare ketones with ambient air. *RSC Adv.* **2023**, 13, 7168-7178.

[2] Wu, H.; Sumita, A.; Otani, Y.; Ohwada, T. Friedel–Crafts Acylation of Aminocarboxylic Acids in Strong Brønsted Acid Promoted by Lewis Base P4O10. *J. Org. Chem.* **2022**, *87*, 15224-15249. DOI: 10.1021/acs.joc.2c01761.

[3] Ding, J.; Luo, S.; Xu, Y.; An, Q.; Yang, Y.; Zuo, Z. Selective oxidation of benzylic alcohols via synergistic bisphosphonium and cobalt catalysis. *Chem. Commun.* **2023**, *59*, 4055-4058, DOI: 10.1039/D3CC00532A.

[4] Wang, M.; Fan, Q.; Jiang, X. Metal-free construction of primary sulfonamides through three diverse salts. *Green Chem,* **2018**, *20*, 5469-5473, DOI: 10.1039/C8GC03014F.

[5] Jiao, J.; Zhang, T.; Xu, J.; Guo, K.; Li, J.; Han, Q. Hydroxyl radical-dominated selective oxidation of ethylbenzene over a photoactive polyoxometalate-based metal–organic framework. *Chem. Commun.* **2023**, 59, 3114-3117, DOI: 10.1039/D2CC06403K.

[6] Bensberg, K.; Savvidis, A.; Ballaschk, F.; Gómez-Suárez, A.; Kirsch, S. F. Oxidation of Alcohols in Continuous Flow with a Solid Phase Hypervalent Iodine Catalyst. *Chem.Eur. J.* **2024**, *30, e202304011*. DOI: 10.1002/chem.202304011.

[7] Moriyama, K.; Takemura, M.; Togo, H. Direct and Selective Benzylic Oxidation of Alkylarenes via C–H Abstraction Using Alkali Metal Bromides. *Org. Lett.* **2012**, *14*, 2414-2417. DOI: 10.1021/ol300853z.

[8] Iyori, Y.; Takahashi, K.; Yamazaki, K.; Ano, Y.; Chatani, N. Nickel-catalyzed reductive defunctionalization of esters in the absence of an external reductant: activation of C–O bonds. *Chem. Commun.* **2019**, *55*, 13610-13613, DOI: 10.1039/C9CC07710C.

NMR Data

Benzophenone (2a). $CDCl_3$ 400 MHz and 101 MHz

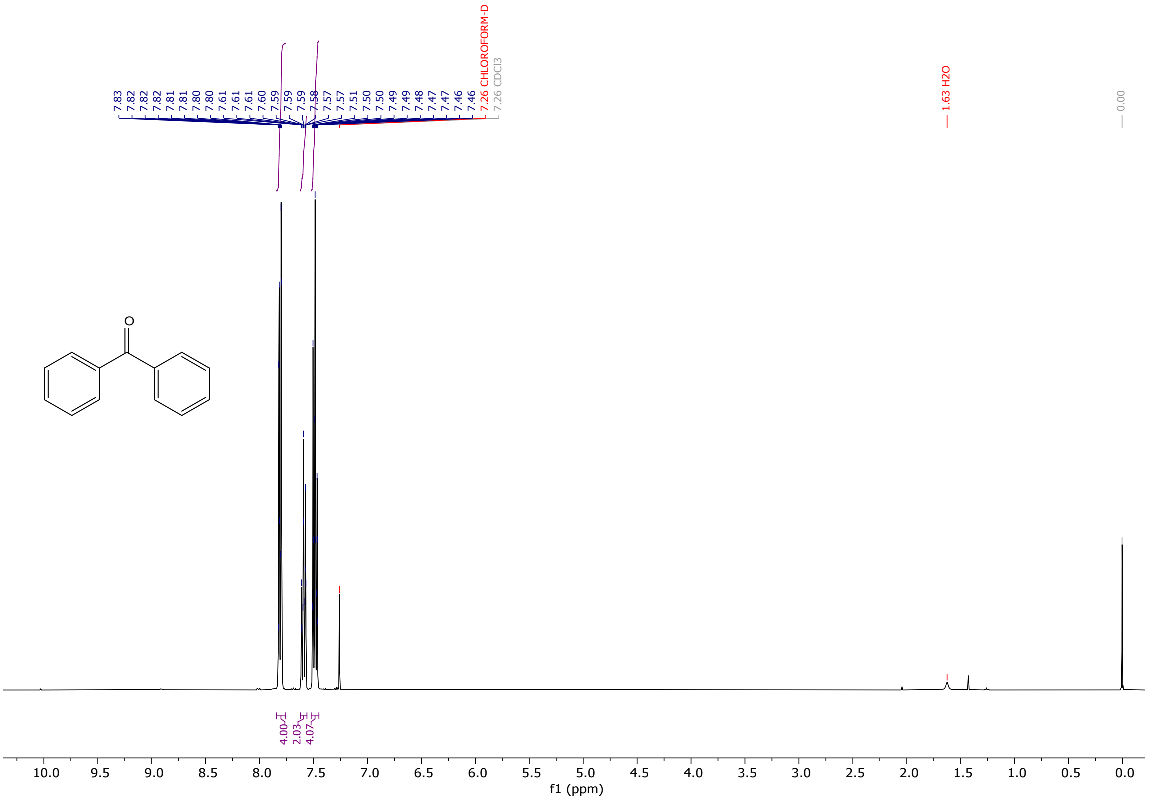

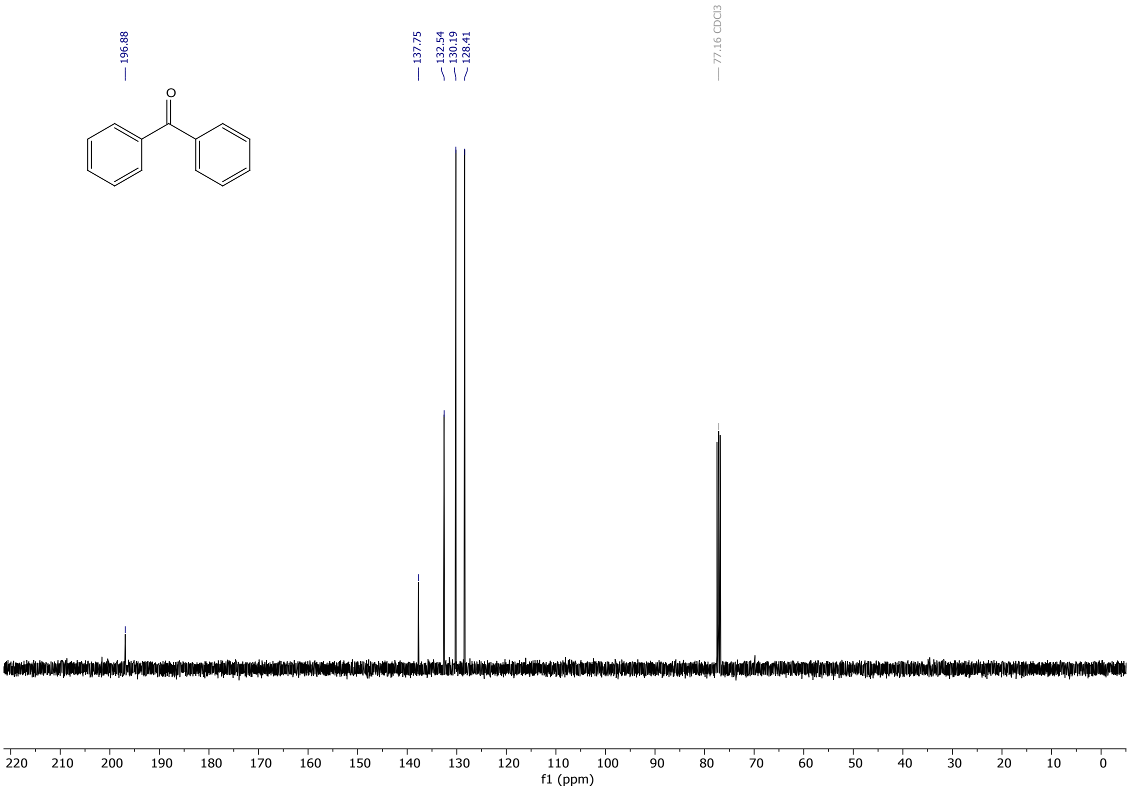

(4-chlorophenyl)(phenyl)methanone (2b). $CDCl_3$ 400 MHz and 101 MHz

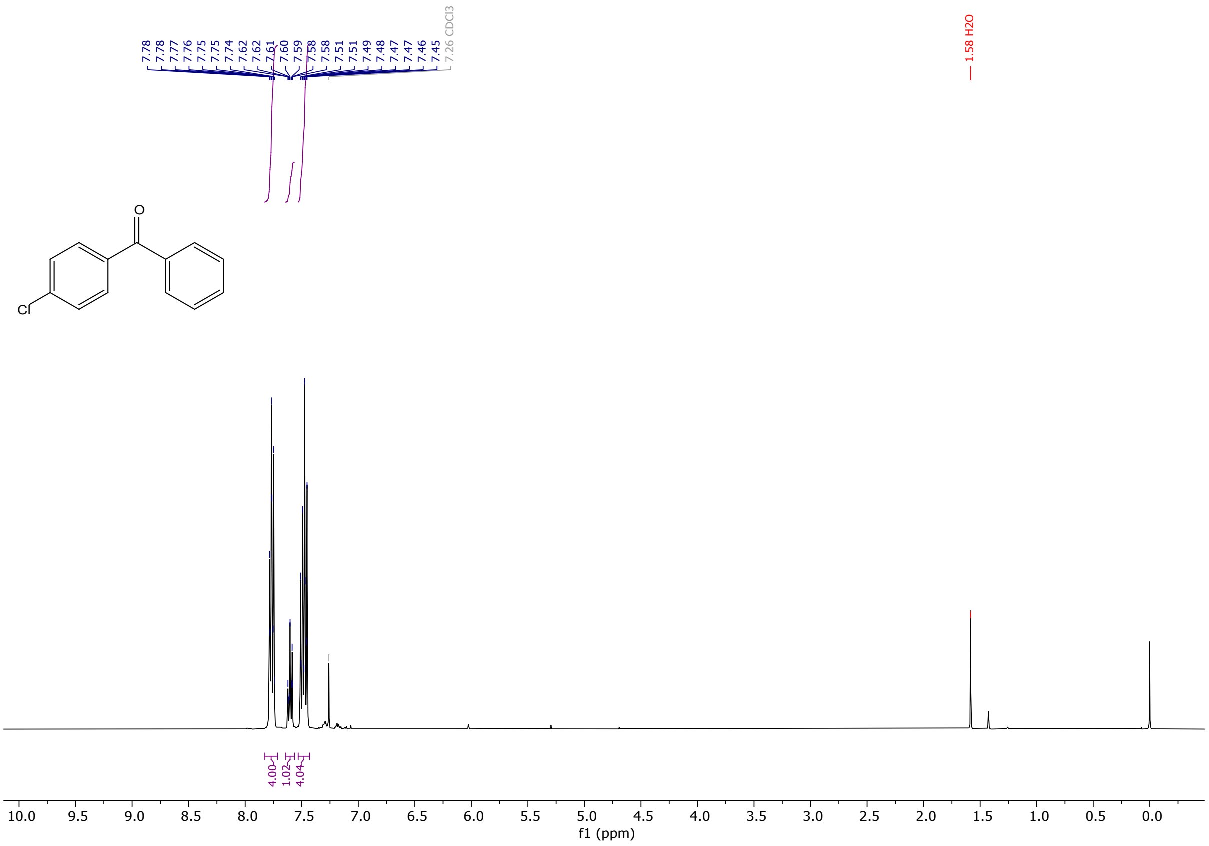

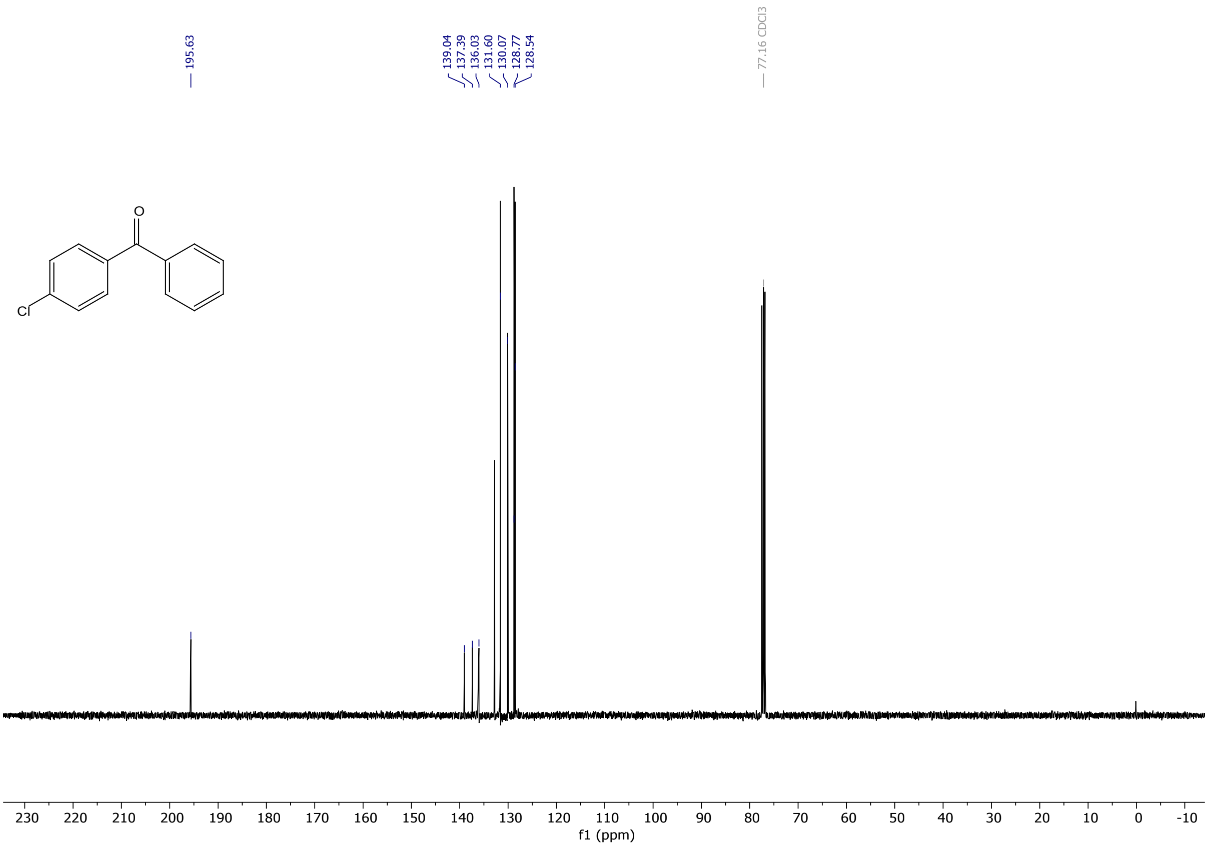

4-Methoxyacetophenone (2c). $CDCl_3$ 400 MHz and 101 MHz

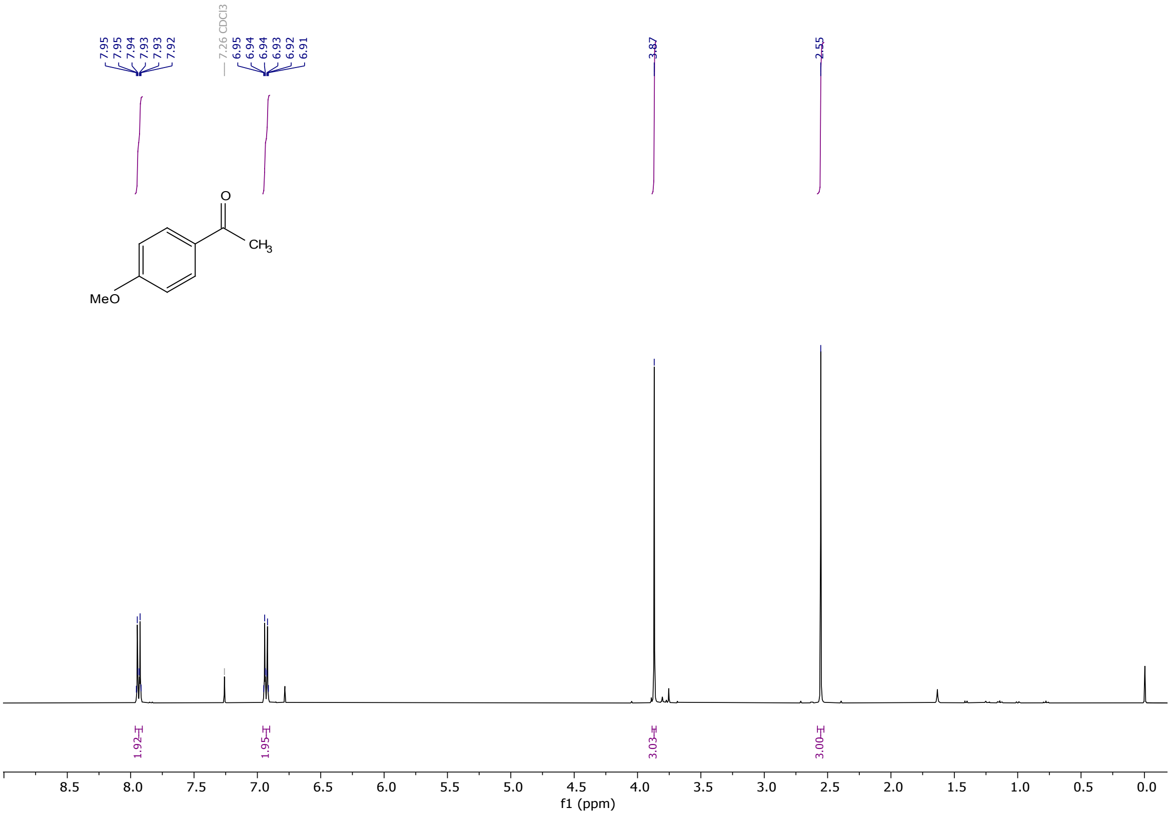

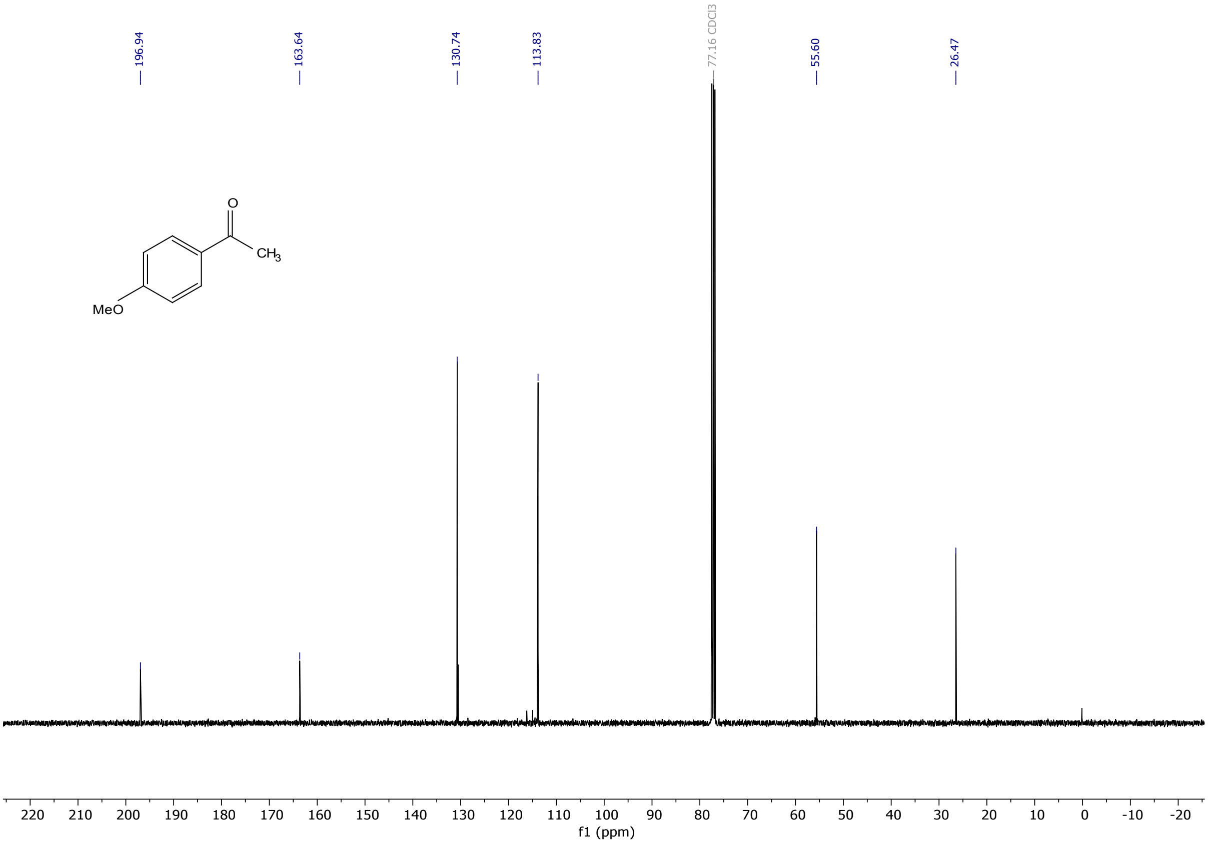

4-Bromoacetophenone (2d). $CDCl_3$ 400 MHz and 101 MHz

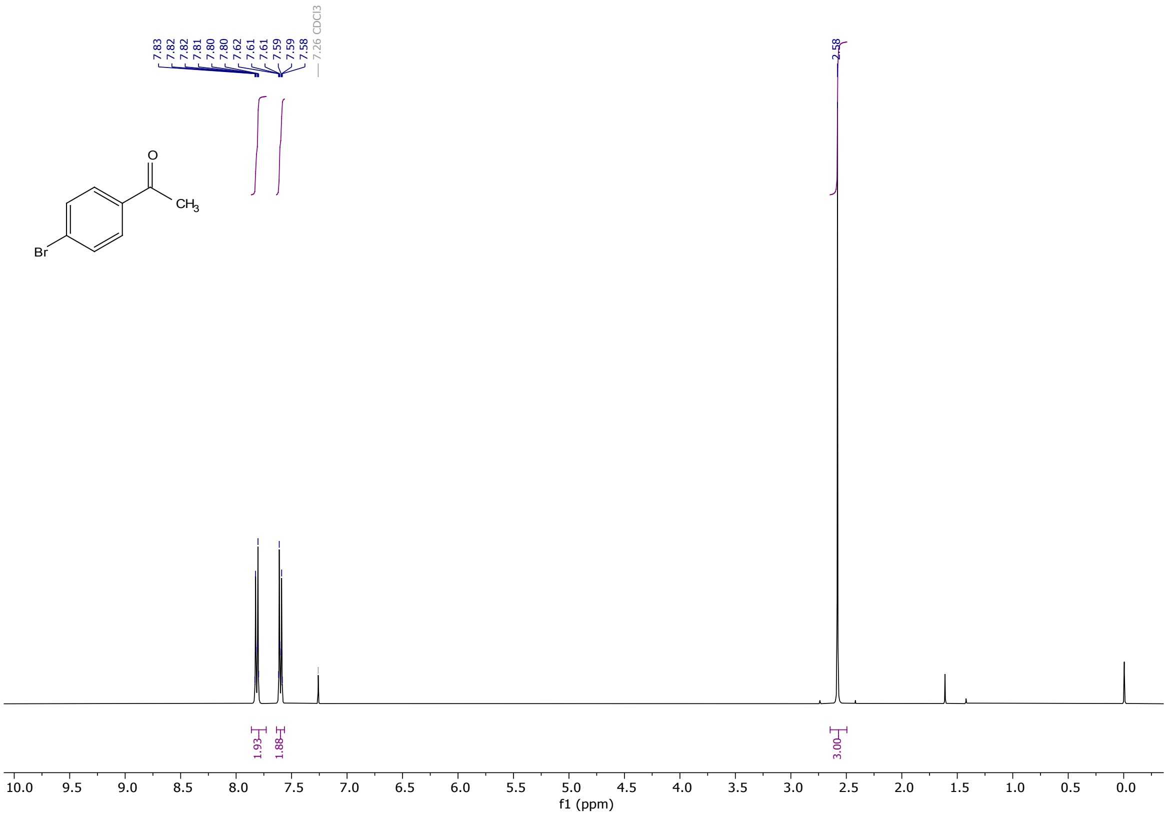

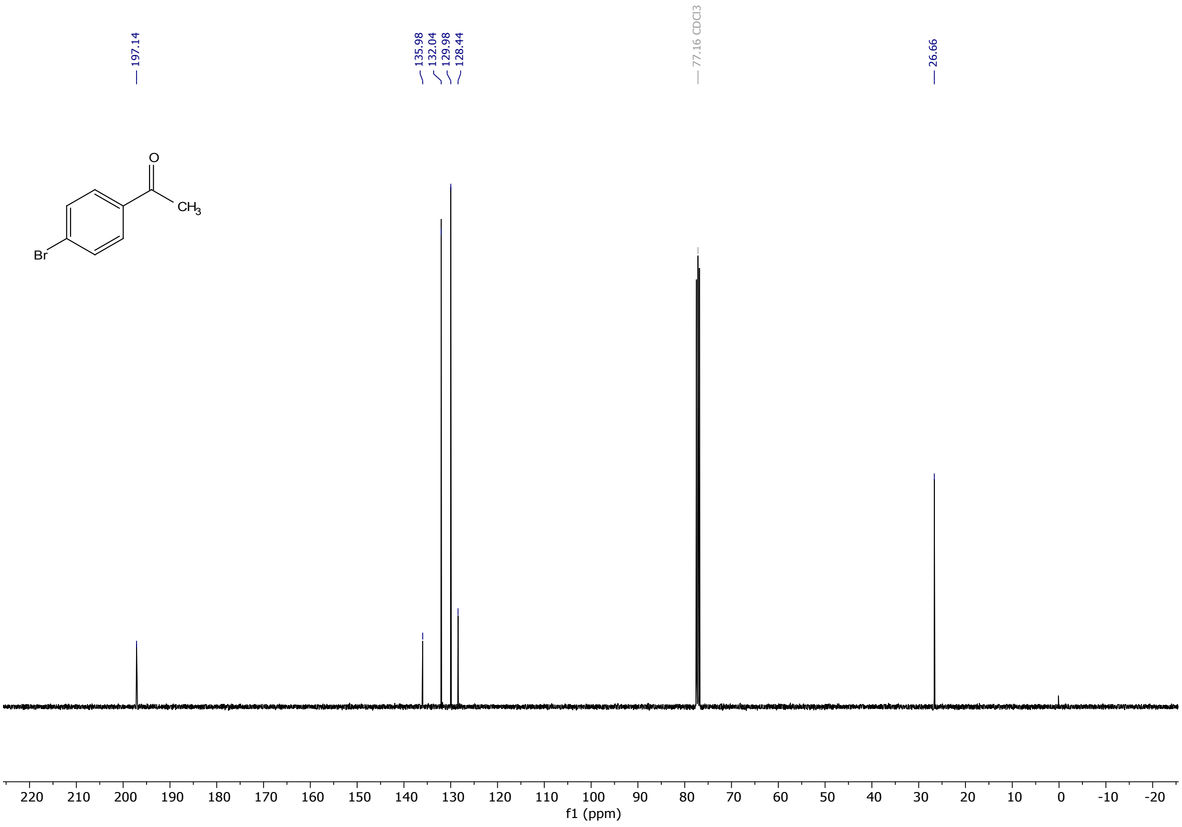

4-Acetylbenzenesulfonamide (2e). DMSO-d_6, 400 MHz and 101 MHz

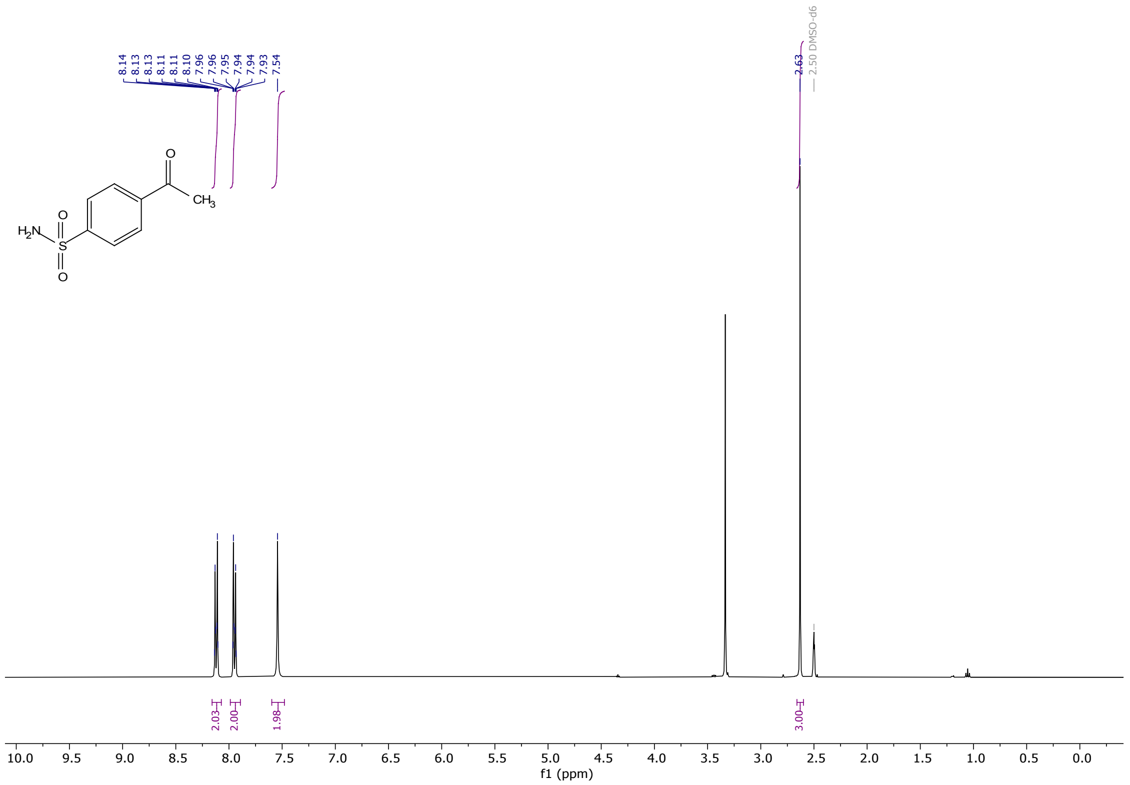

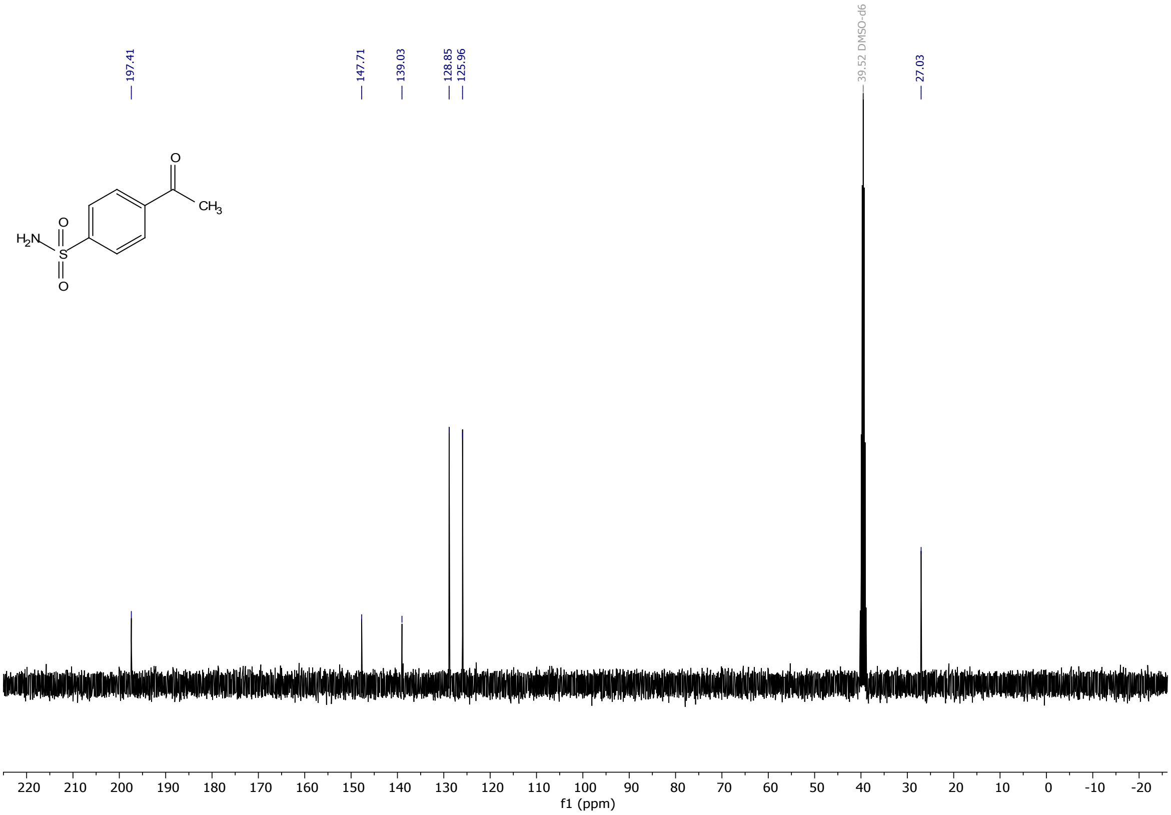

Acetophenone (2f). $CDCl_3$ 400 MHz and 101 MHz

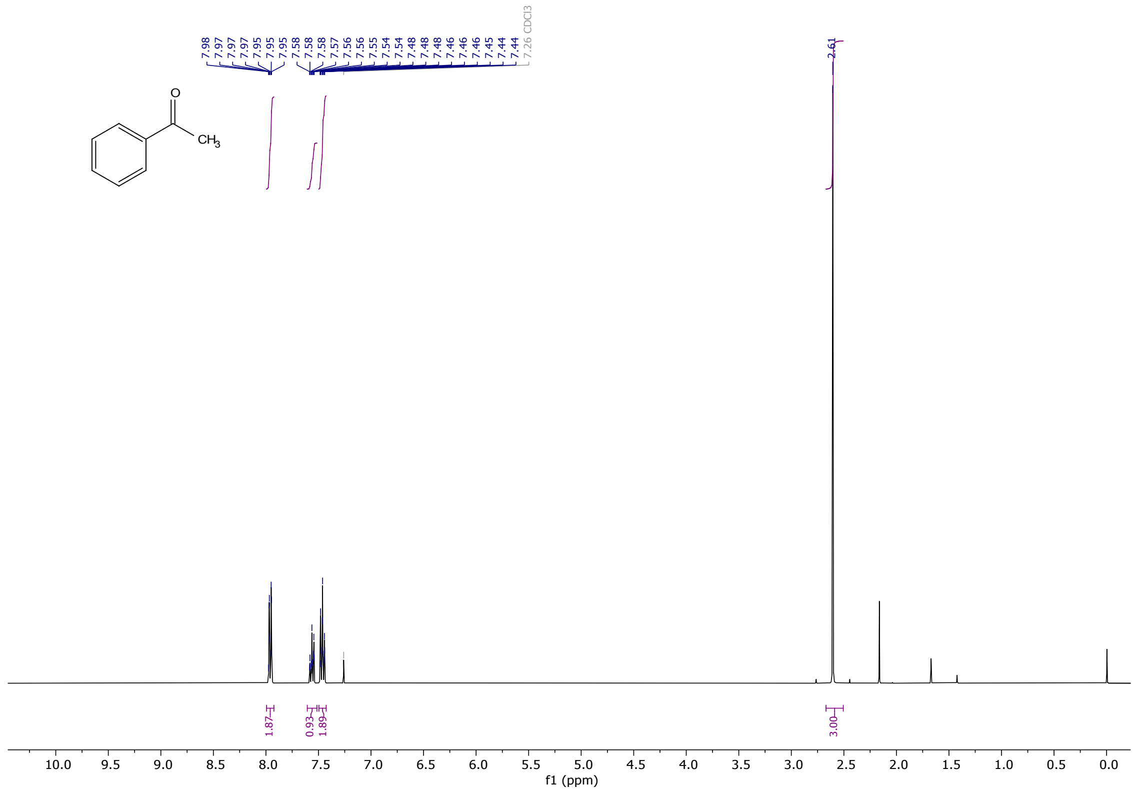

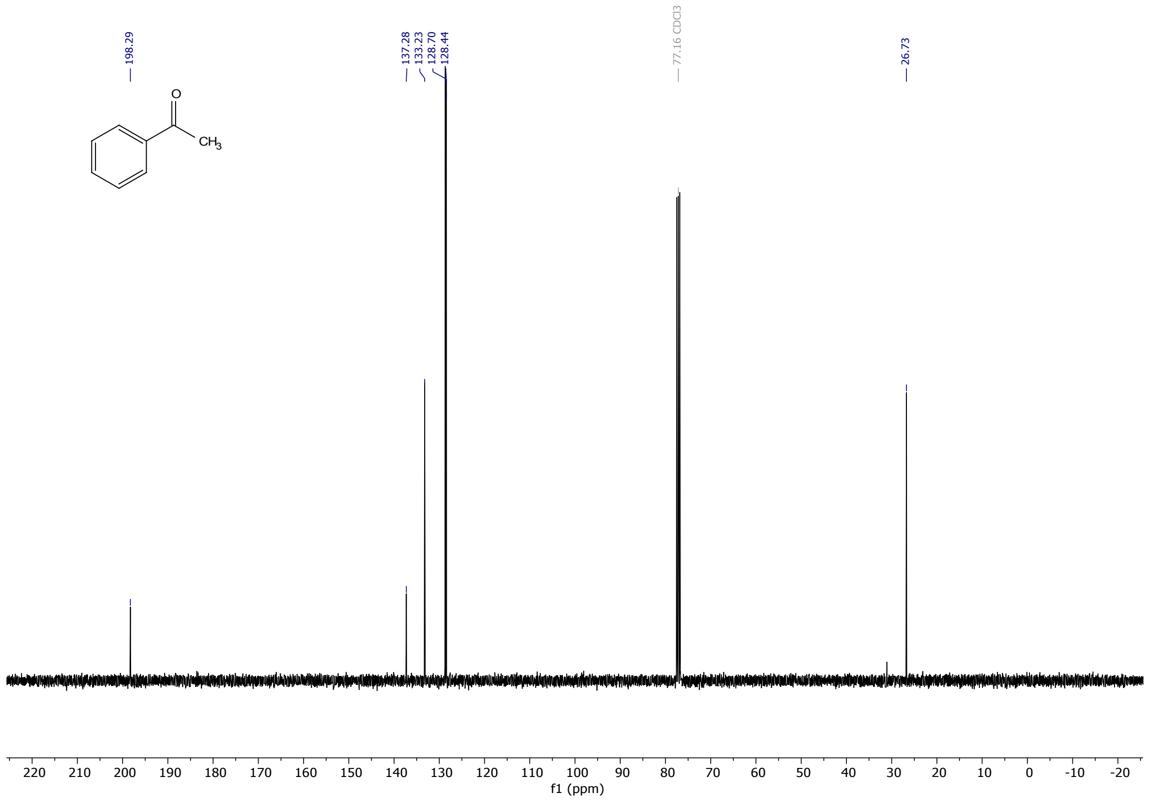

2,3-Dihydro-1H-inden-1-one (2g). $CDCl_3$ 400 MHz and 101 MHz

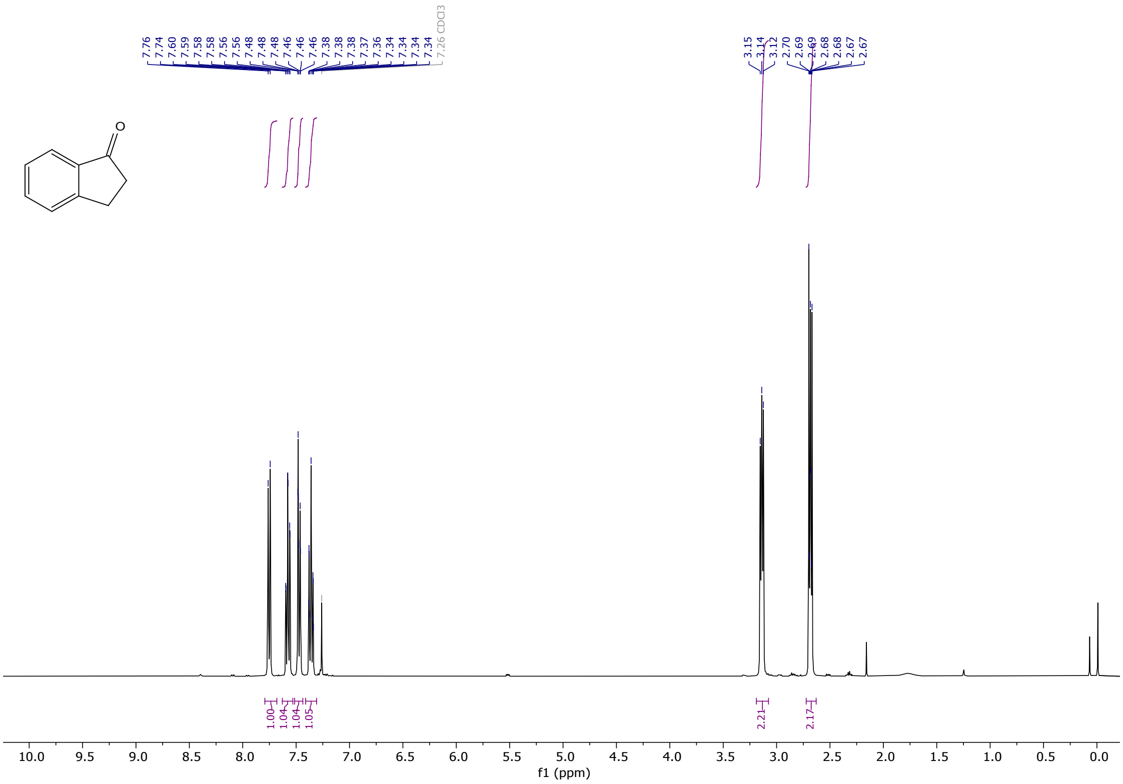

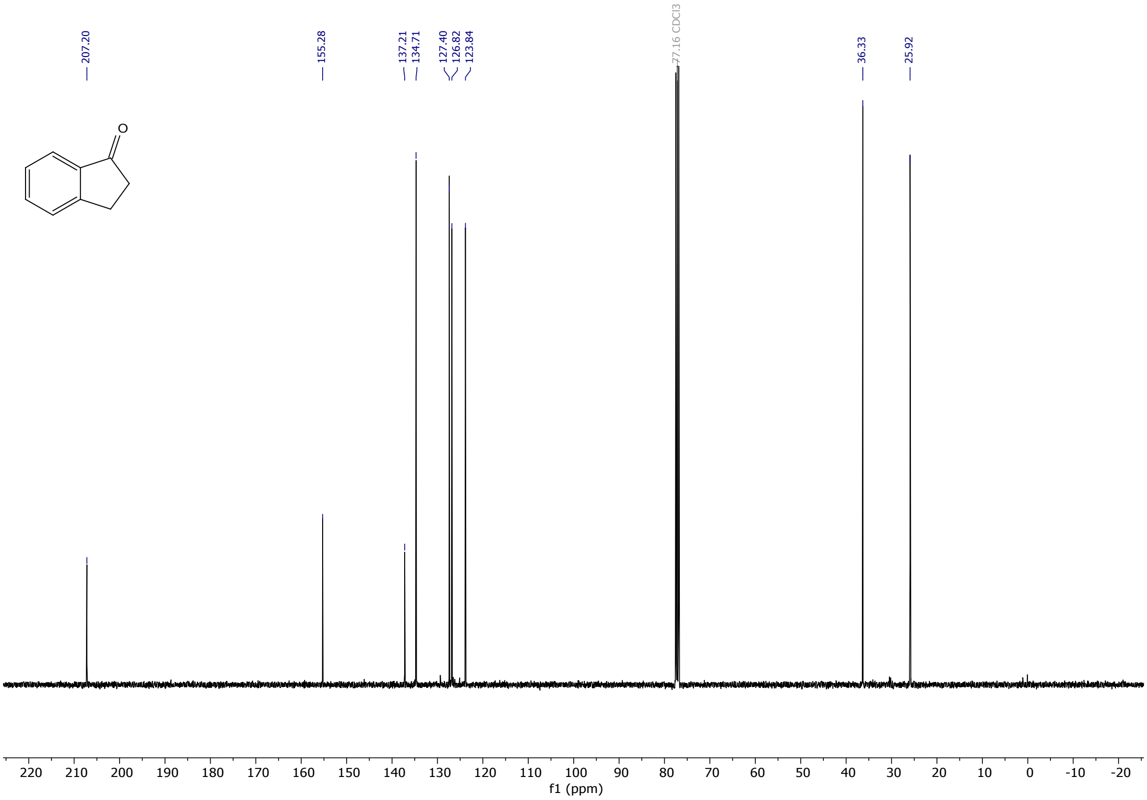

3-Hydroxy-1-phenylpropan-1-one (2h). $CDCl_3$ 400 MHz and 101 MHz

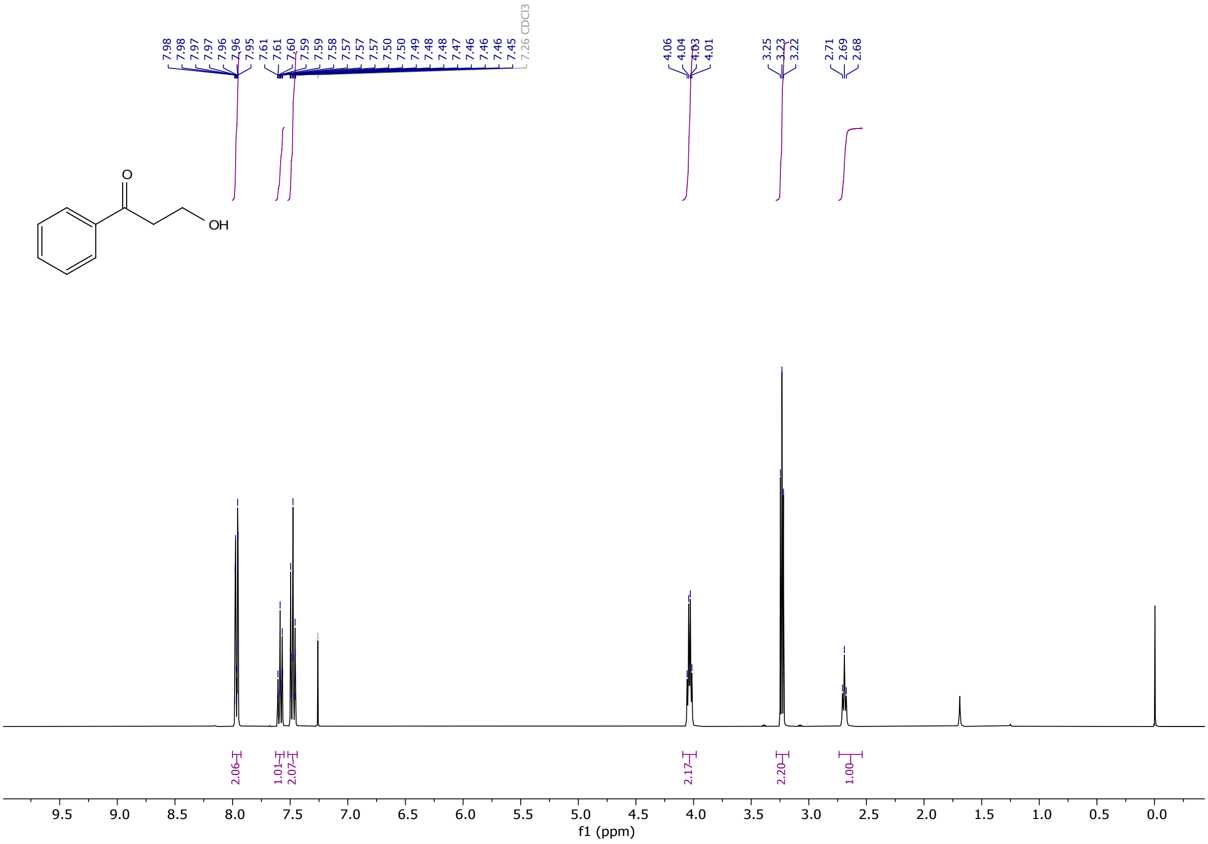

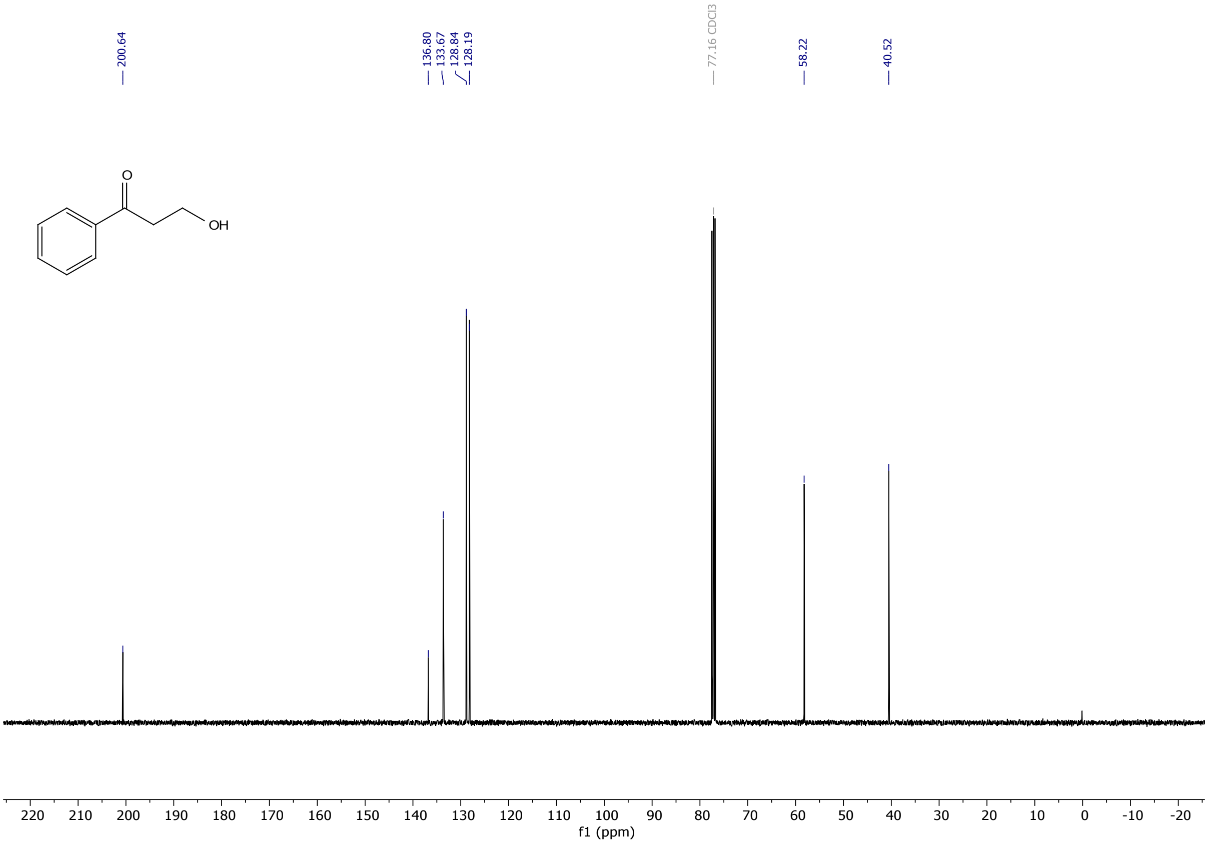

1-(2-Nitrophenyl)ethan-1-one (2i). $CDCl_3$ 400 MHz and 101 MHz

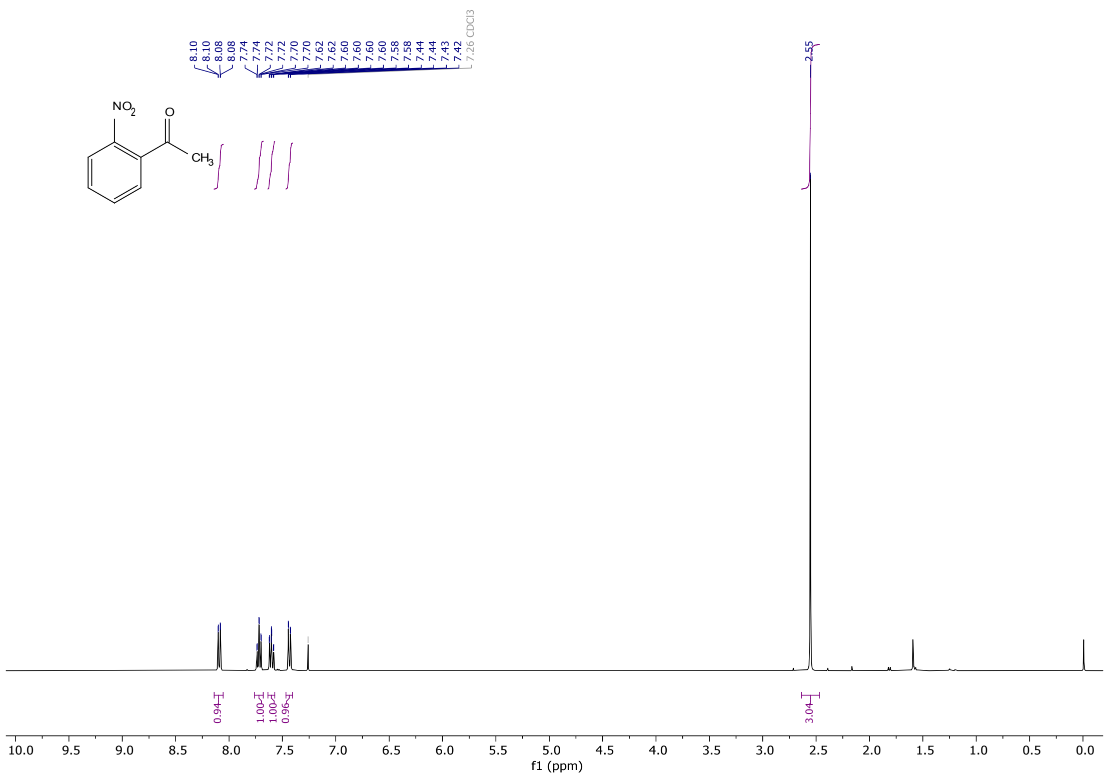

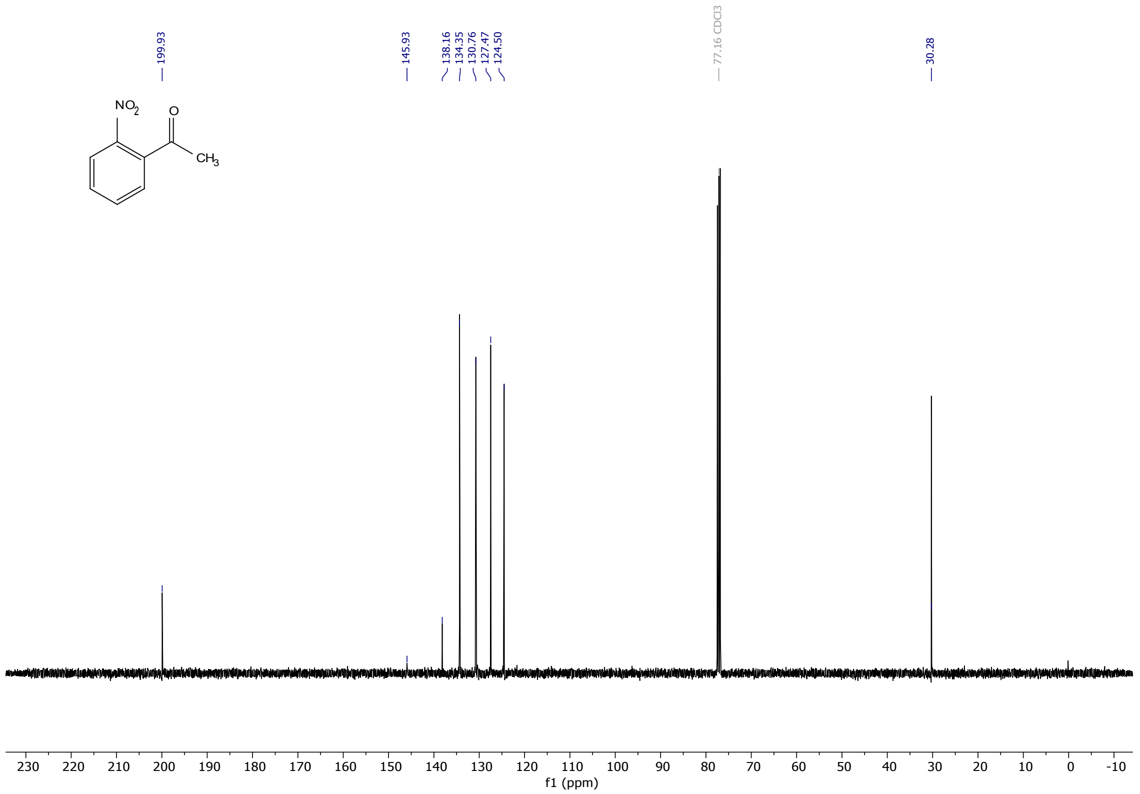

Methyl 2-oxo-2-phenylacetate (2j). $CDCl_3$ 400 MHz and 101 MHz

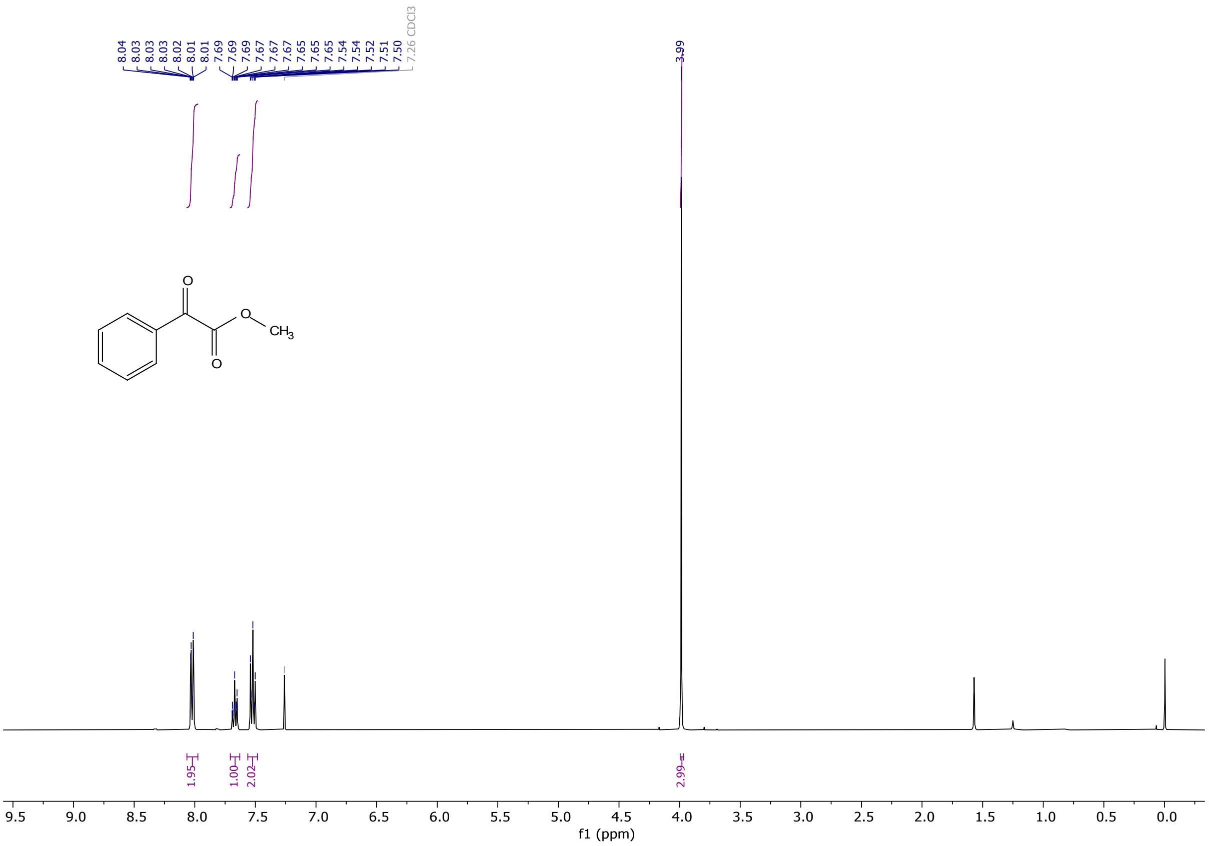

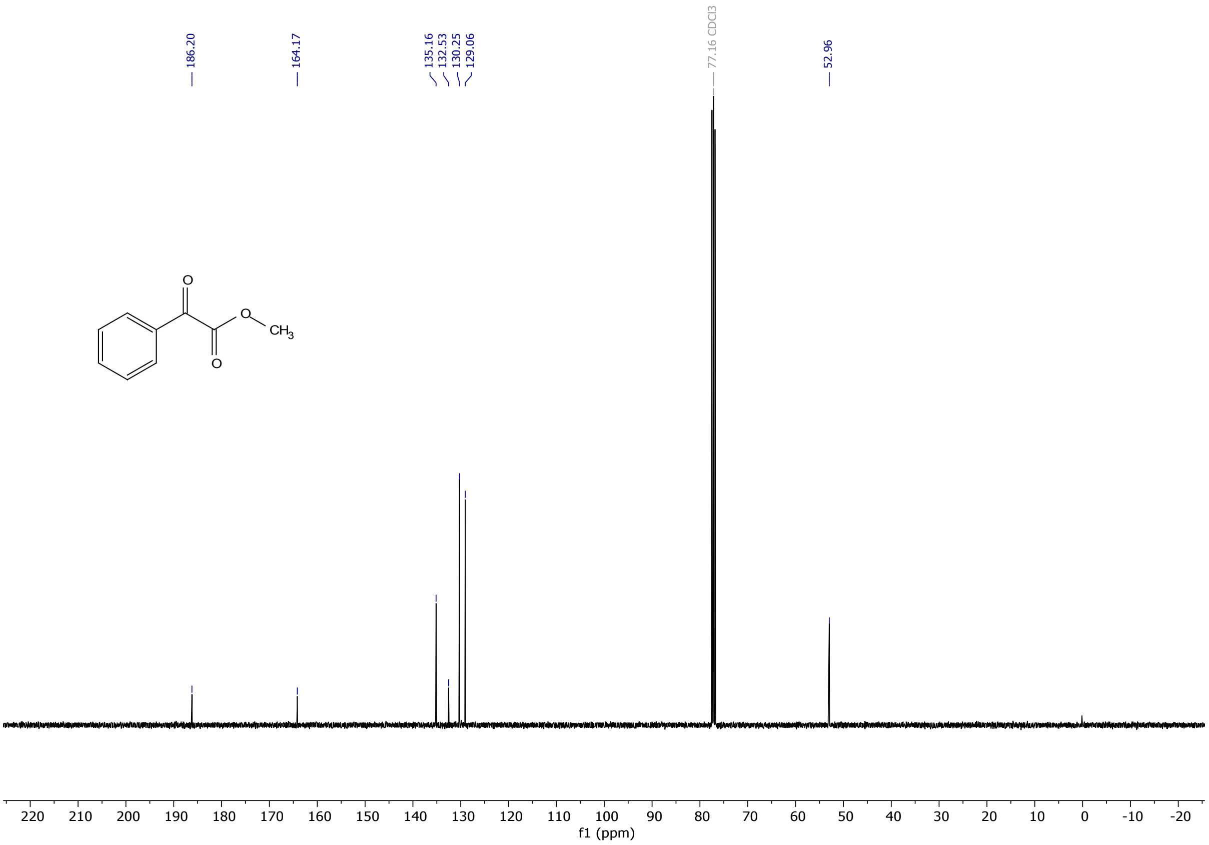

Isobutylphenone (2k). $CDCl_3$ 400 MHz and 101 MHz

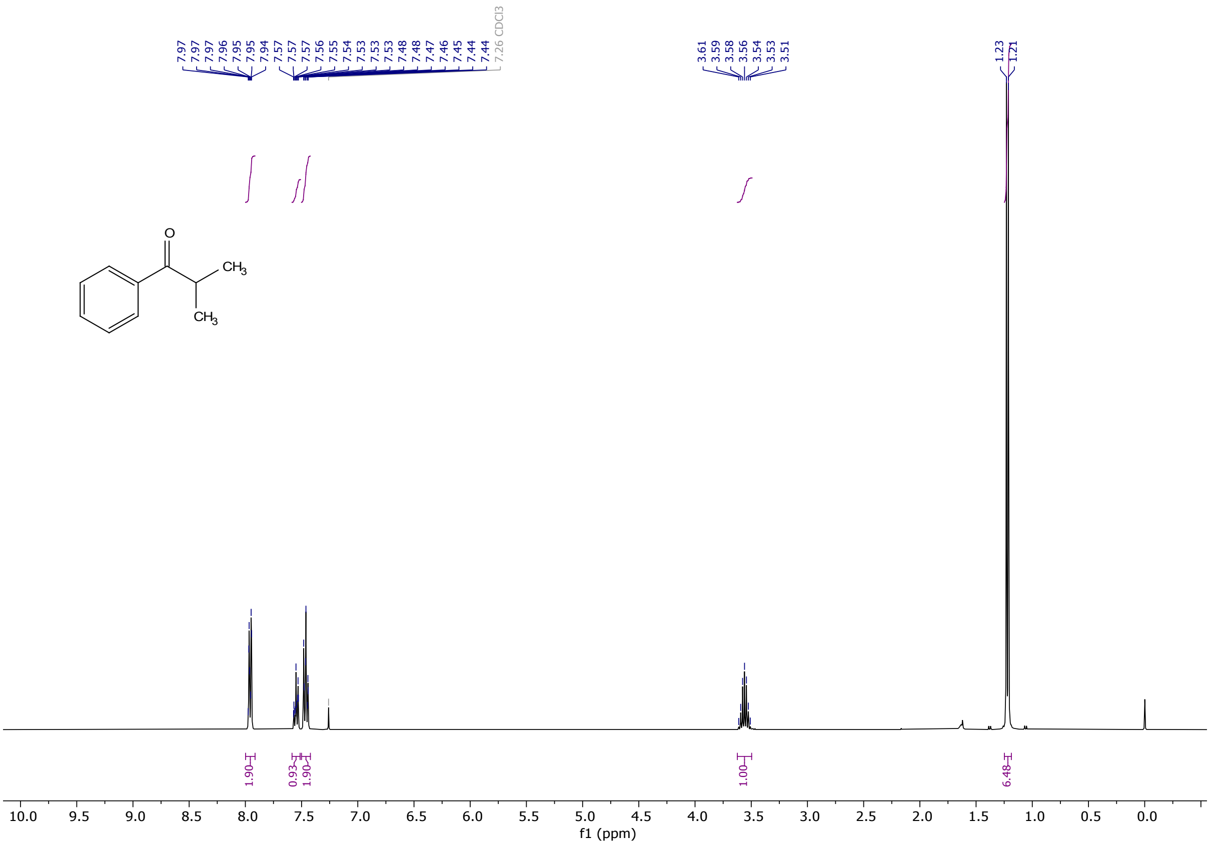

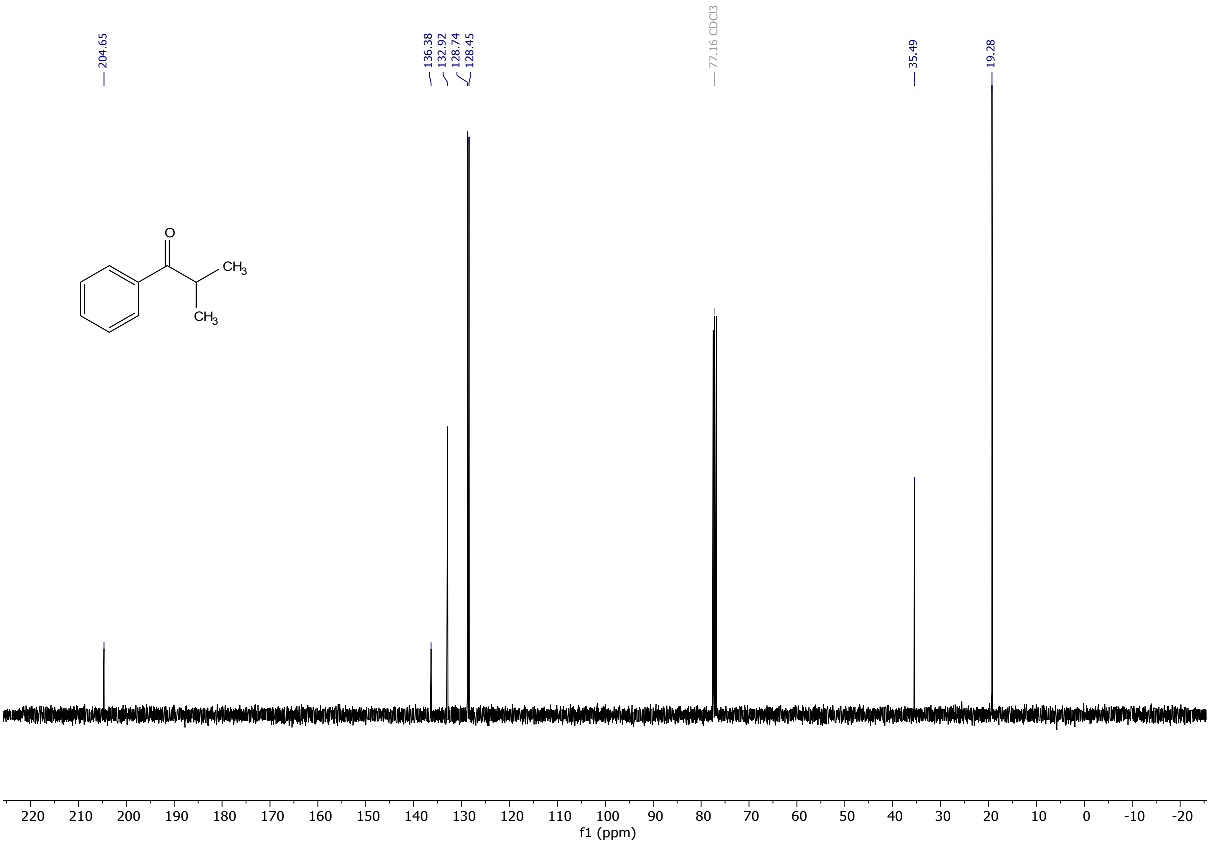

4-Methoxybenzaldeyde (2l). $CDCl_3$ 400 MHz and 101 MHz

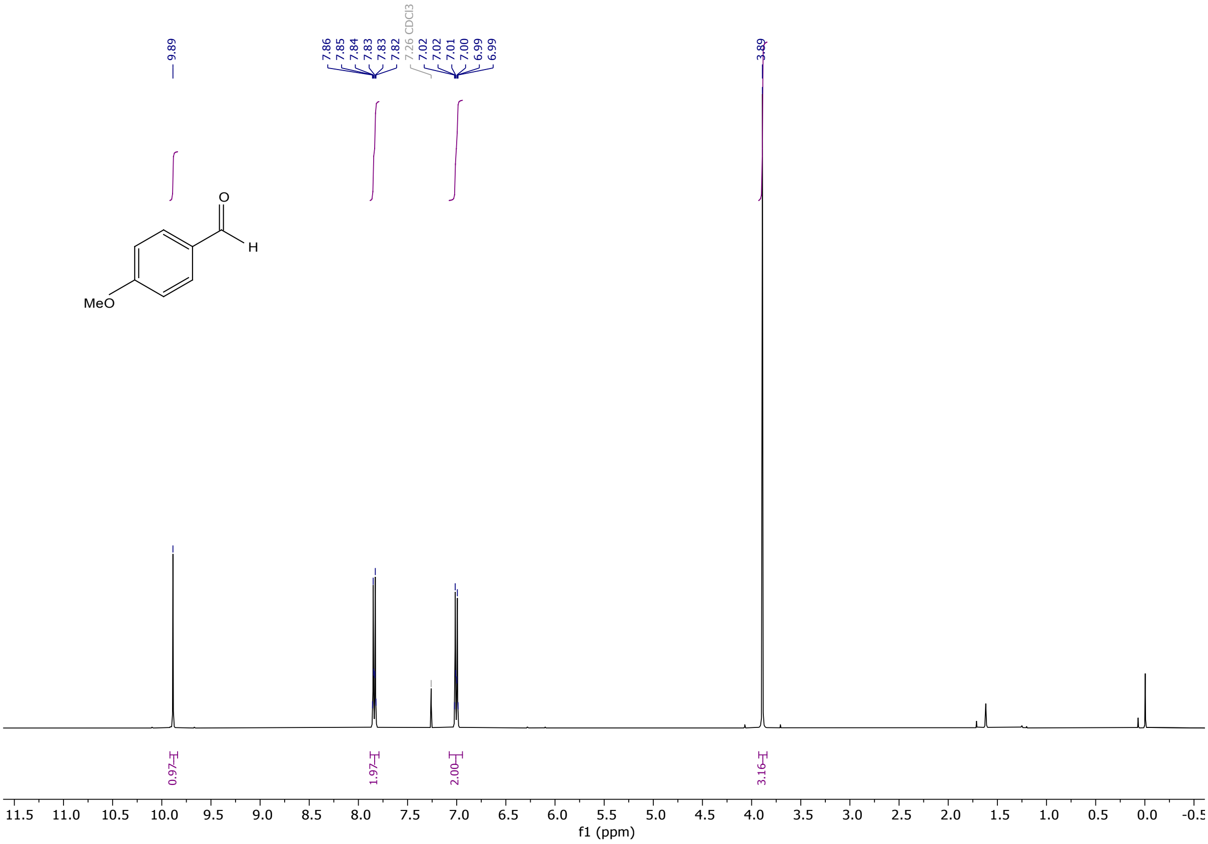

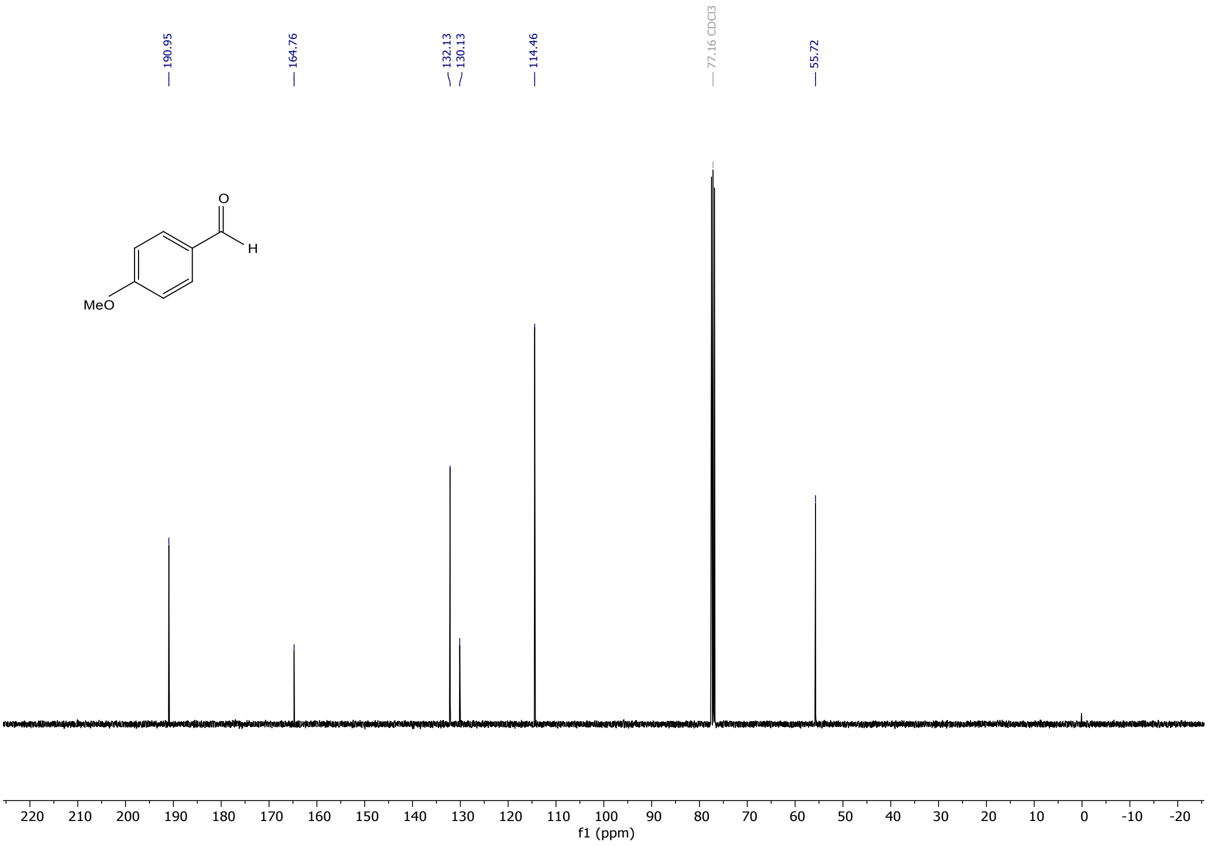

4-Acetylbenzaldehyde (2m). $CDCl_3$ 400 MHz and 101 MHz

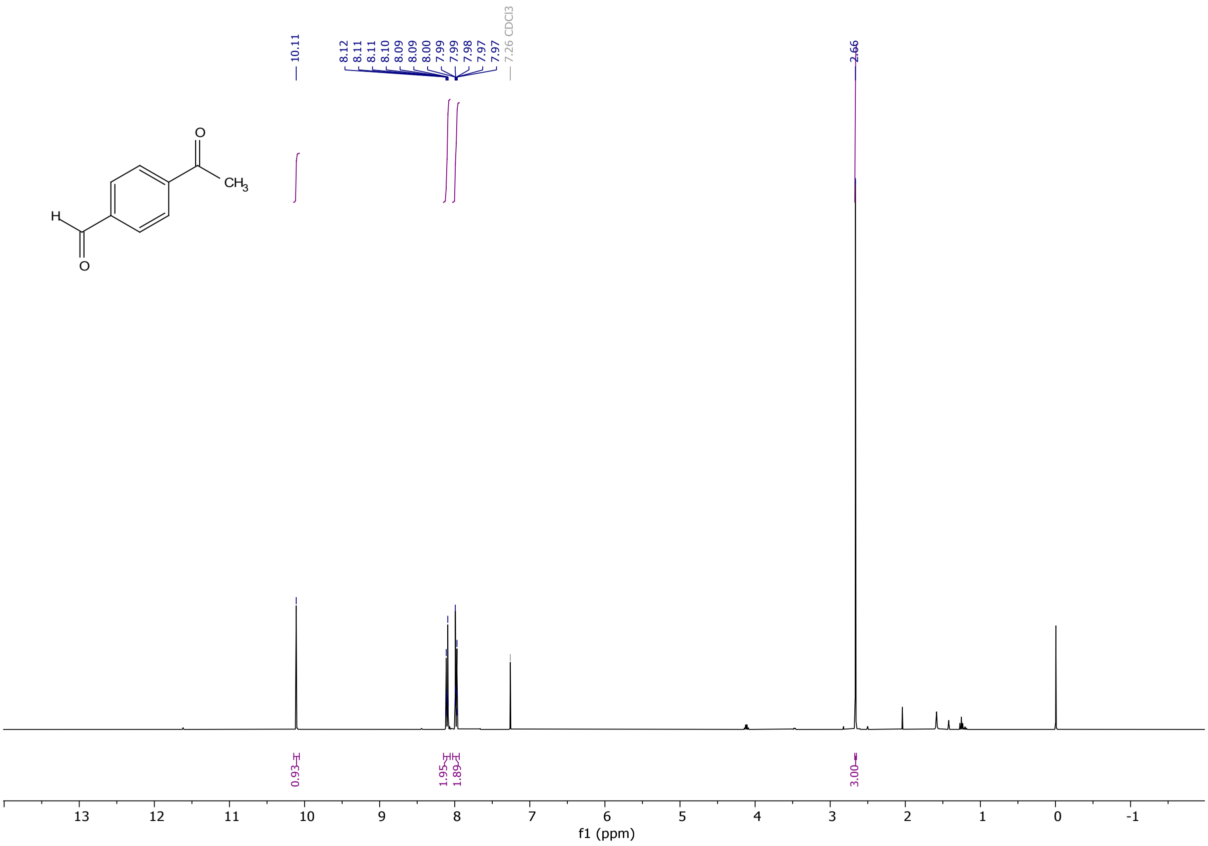

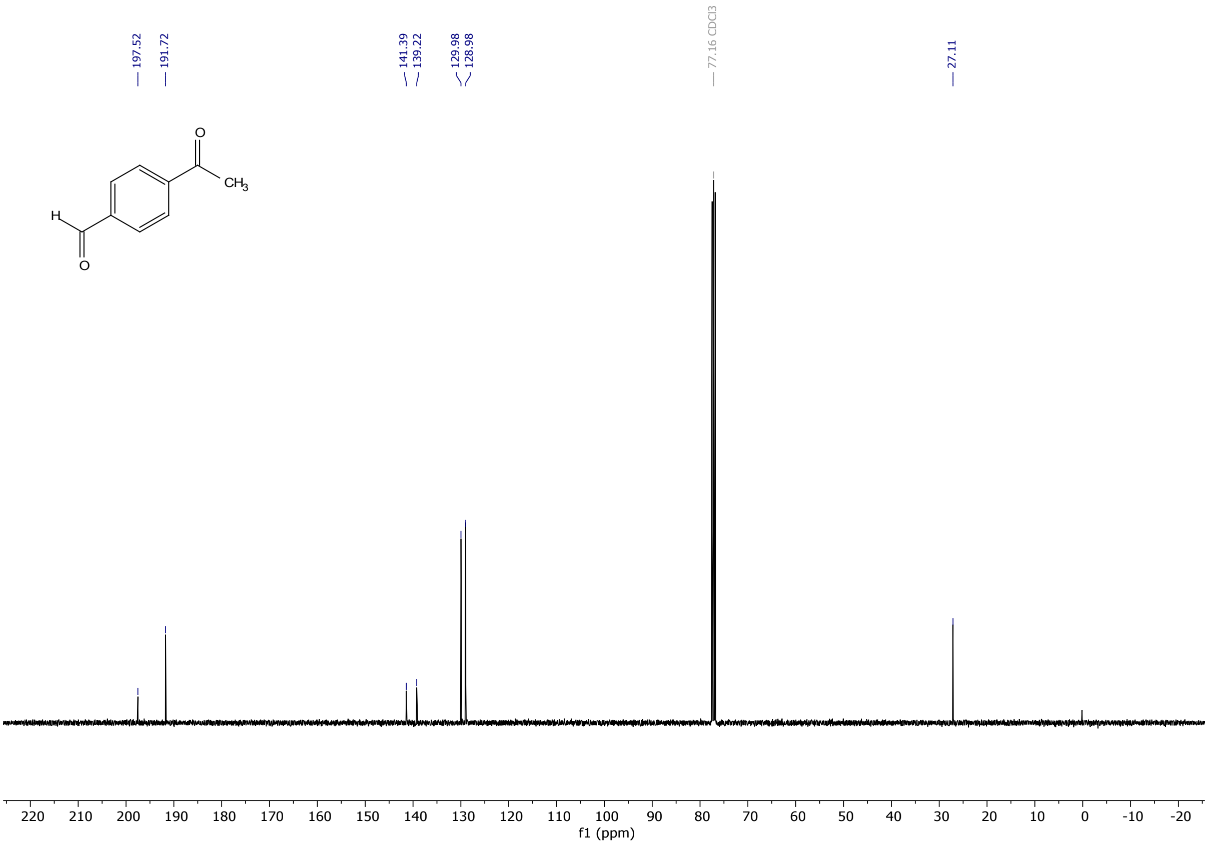

Ethyl 2-formylbenzoate (2n). $CDCl_3$ 400 MHz and 101 MHz

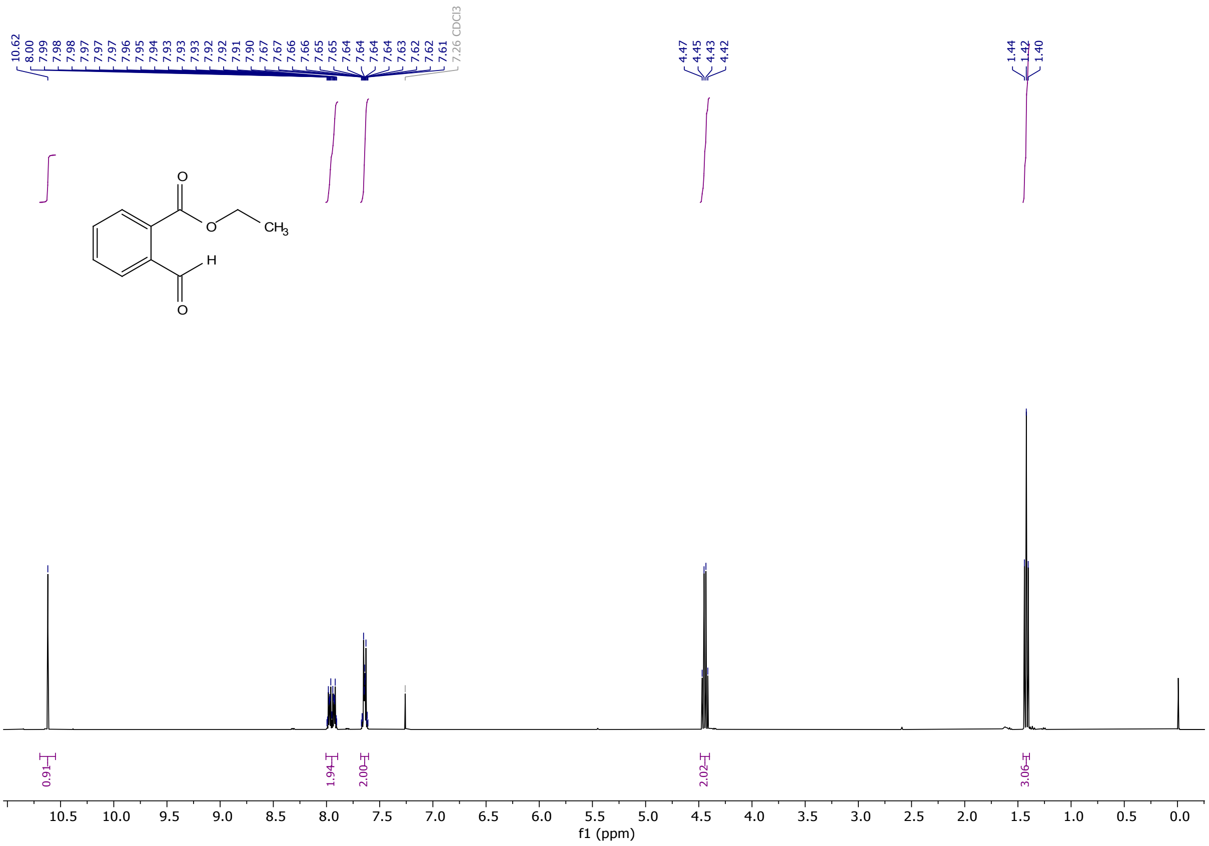

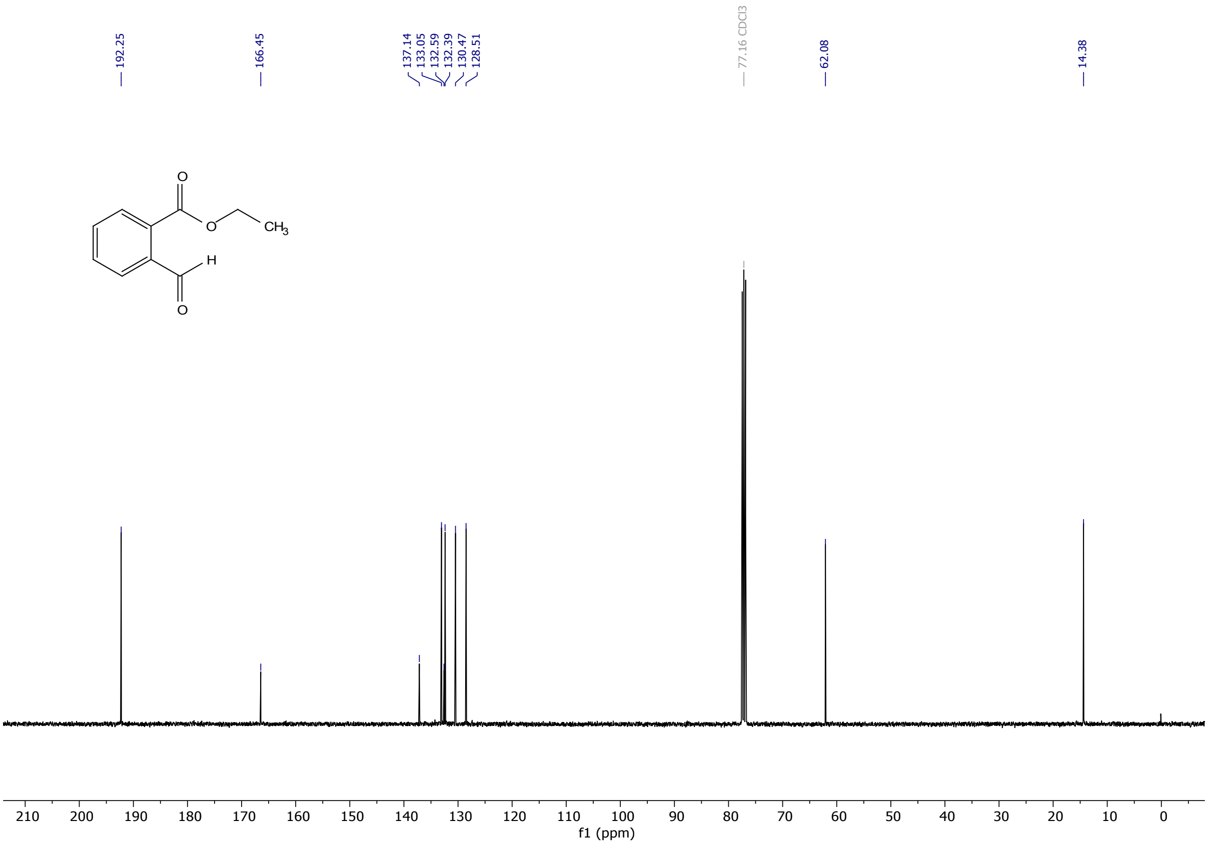

Supplement: Supplementary file 1 — op4c00213_si_001.pdf [file op4c00213_si_001.pdf]
